# Topologically associating domain boundaries are required for normal genome function

**DOI:** 10.1038/s42003-023-04819-w

**Published:** 2023-04-20

**Authors:** Sudha Rajderkar, Iros Barozzi, Yiwen Zhu, Rong Hu, Yanxiao Zhang, Bin Li, Ana Alcaina Caro, Yoko Fukuda-Yuzawa, Guy Kelman, Adyam Akeza, Matthew J. Blow, Quan Pham, Anne N. Harrington, Janeth Godoy, Eman M. Meky, Kianna von Maydell, Riana D. Hunter, Jennifer A. Akiyama, Catherine S. Novak, Ingrid Plajzer-Frick, Veena Afzal, Stella Tran, Javier Lopez-Rios, Michael E. Talkowski, K. C. Kent Lloyd, Bing Ren, Diane E. Dickel, Axel Visel, Len A. Pennacchio

**Affiliations:** 1 Environmental Genomics & System Biology Division, Lawrence Berkeley National Laboratory, 1 Cyclotron Road, Berkeley, CA 94720 USA; 2 Center for Cancer Research, Medical University of Vienna, Borschkegasse 8a, 1090 Vienna, Austria; 3 Department of Surgery and Cancer, Imperial College London, London, UK; 4 Ludwig Institute for Cancer Research, La Jolla, CA USA; 5 Centro Andaluz de Biología del Desarrollo (CABD), CSIC-Universidad Pablo de Olavide Junta de Andalucía, 41013 Seville, Spain; 6 Institute of Advanced Biosciences, Keio University, Tsuruoka, Yamagata Japan; 7 The Jerusalem Center for Personalized Computational Medicine, Hebrew University of Jerusalem, Jerusalem, Israel; 8 U.S. Department of Energy Joint Genome Institute, 1 Cyclotron Road, Berkeley, CA 94720 USA; 9 Center for Genomic Medicine, Massachusetts General Hospital, Boston, MA 02114 USA; 10 Program in Medical and Population Genetics and Stanley Center for Psychiatric Disorders, Broad Institute of Harvard and Massachusetts Institute of Technology, Cambridge, MA 02142 USA; 11 Department of Neurology, Massachusetts General Hospital and Harvard Medical School, Boston, MA 02114 USA; 12 Mouse Biology Program, University of California, Davis, Davis, CA USA; 13 Department of Surgery, School of Medicine, University of California, Davis, Davis, CA USA; 14 Center for Epigenomics, University of California, San Diego School of Medicine, La Jolla, CA USA; 15 Department of Cellular and Molecular Medicine, University of California, San Diego School of Medicine, La Jolla, CA USA; 16 Institute of Genome Medicine, Moores Cancer Center, University of California, San Diego School of Medicine, La Jolla, CA USA; 17 School of Natural Sciences, University of California, Merced, Merced, CA USA; 18 Comparative Biochemistry Program, University of California, Berkeley, CA 94720 USA

**Keywords:** Epigenomics, Epigenomics

## Abstract

Topologically associating domain (TAD) boundaries partition the genome into distinct regulatory territories. Anecdotal evidence suggests that their disruption may interfere with normal gene expression and cause disease phenotypes^1–3^, but the overall extent to which this occurs remains unknown. Here we demonstrate that targeted deletions of TAD boundaries cause a range of disruptions to normal in vivo genome function and organismal development. We used CRISPR genome editing in mice to individually delete eight TAD boundaries (11–80 kb in size) from the genome. All deletions examined resulted in detectable molecular or organismal phenotypes, which included altered chromatin interactions or gene expression, reduced viability, and anatomical phenotypes. We observed changes in local 3D chromatin architecture in 7 of 8 (88%) cases, including the merging of TADs and altered contact frequencies within TADs adjacent to the deleted boundary. For 5 of 8 (63%) loci examined, boundary deletions were associated with increased embryonic lethality or other developmental phenotypes. For example, a TAD boundary deletion near *Smad3*/*Smad6* caused complete embryonic lethality, while a deletion near *Tbx5*/*Lhx5* resulted in a severe lung malformation. Our findings demonstrate the importance of TAD boundary sequences for in vivo genome function and reinforce the critical need to carefully consider the potential pathogenicity of noncoding deletions affecting TAD boundaries in clinical genetics screening.

## Abstract (web summary)

Targeted deletions of topologically associating domain boundaries in mice using CRISPR genome editing cause a range of disruptions to normal in vivo genome function and organismal development.

## Introduction

Eukaryotic genomes fold into topologically associating domains (TADs), sub-megabase-scale chromatin segments characterized by high intra-domain chromatin contact frequency^4–6^. TADs represent a key feature of hierarchical genome organization by defining chromatin neighborhoods within which regulatory sequences can interact, while simultaneously insulating regulatory interactions across boundaries^5,7–10^. TAD boundaries are primarily defined and measured through chromatin conformation assays, and they are typically associated with a signature set of proteins, including CCCTC-binding factor (CTCF), components of the structural maintenance of chromosomes (SMC) complex such as cohesin and condensin, and RNA polymerase II^5,11–14^. TADs form as a result of loop extrusion, wherein DNA strands slide from within the cohesin or SMC complex until bound CTCF molecules in a convergent orientation are met^13,15–19^. Loss of CTCF, cohesin, or the cohesin loading factor Nipbl results in TAD disruption, while loss of cohesin release factor, Wapl, results in reinforcement of TAD boundaries^20,21^. Intriguingly, ~20% of TAD boundaries remain stable upon loss of CTCF^22^. Both CTCF-mediated mechanisms and transcription can affect the formation and function of TADs, but neither seems to be individually sufficient nor universally required^7,11,23,24^. Thus, chromatin state, transcriptional activity, and TAD organization may influence each other, and the observed nuclear structure of mammalian genomes likely results from their complex interplay^7,11,23–25^.

The genomic locations of TAD boundaries are well conserved across mammalian species, indicating that their function and positions within the genome are subject to evolutionary constraint^5,26–28^. This notion is further supported by the overall depletion of structural variants at TAD boundaries observed in the general human population^26^, while disruptions and rearrangements of TAD structure have been implicated in the mis-expression of genes and are associated with developmental and cancer phenotypes^1–3,29,30^. However, most of these disruptions were spontaneously occurring large structural mutations that also included neighboring genomic features, such as regulatory elements and/or protein-coding genes. Therefore, the specific role of TAD boundaries in these phenotypes is not well understood. In the present study, we examine the functional necessity of TAD boundary sequences in vivo. We selected eight independent TAD boundaries in the vicinity of genes active during embryonic development, individually deleted these boundaries from the mouse genome, and systematically examined the consequences on survival, genome organization, gene expression, and development. All eight TAD boundary deletions caused alterations of one or more of these properties. We also observed that loss of boundaries with more CTCF sites generally resulted in more severe phenotypes and that the most severe organismal phenotypes coincided with pronounced changes in chromatin conformation. In combination, our results indicate that TAD boundary sequences are required for normal genome function and development.

## Results

### Strategy for selecting TAD boundaries for in vivo deletion

**Fig. 1:**
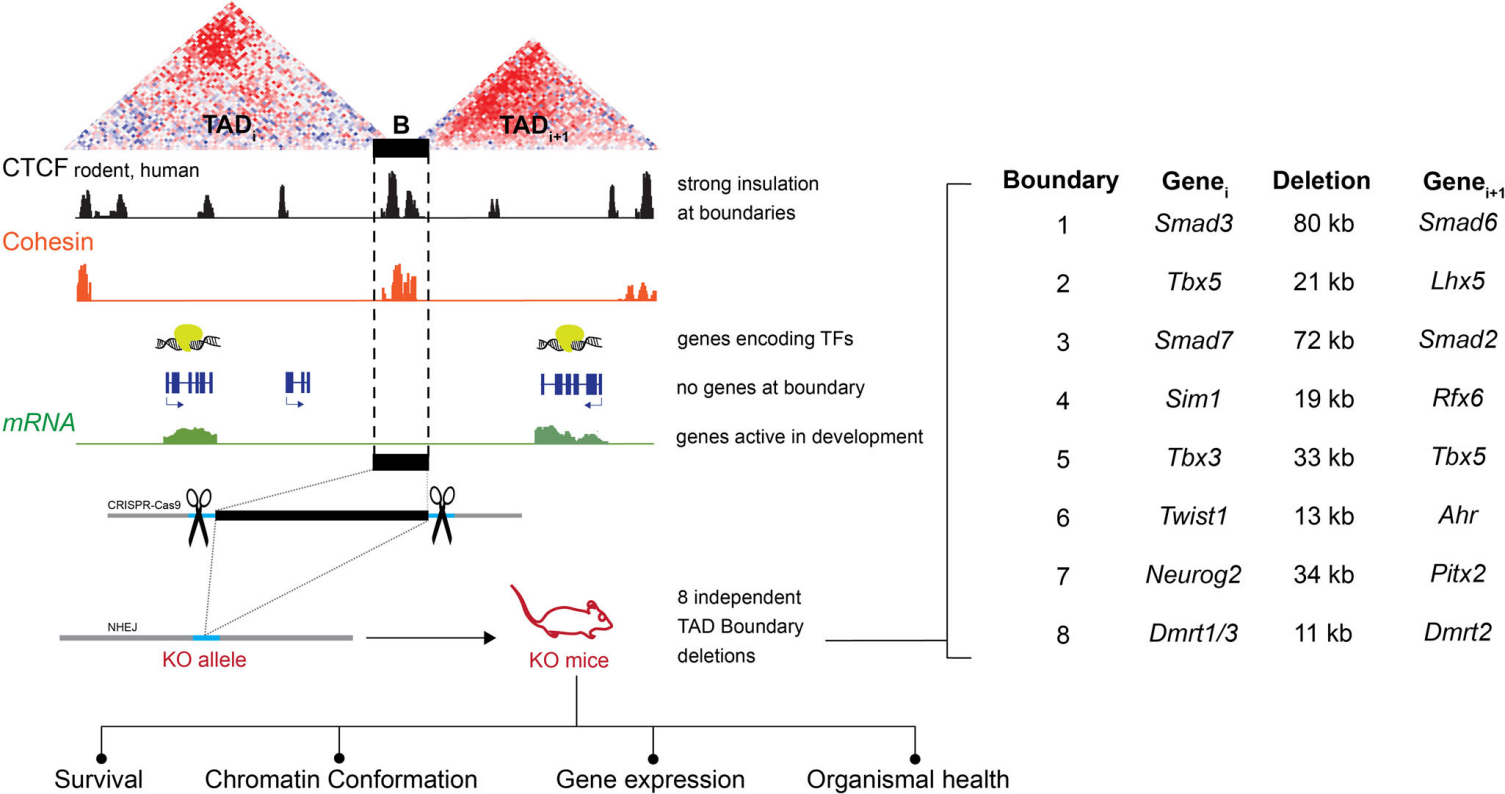
Study overview. Schematic showing the selection and CRISPR/Cas9-based deletion strategy for removal of TAD boundaries in vivo, along with types of phenotyping performed on the resulting knockout (KO) mice. Specific boundaries individually deleted, along with selected developmentally expressed genes flanking each deleted boundary are also depicted. B boundary element, TFs transcription factors.

To assess the in vivo functions of TAD boundary sequences, we focused on boundaries flanking TADs that harbor genes with known expression and function during embryonic development to facilitate the detection of phenotypes resulting from boundary deletion. From a genome-wide set of >3300 previously annotated TAD boundaries^5,10^, we scored and prioritized each boundary based on the following criteria: (1) CTCF occupancy aggregated from 62 published CTCF ChIP-seq datasets, which served as a proxy for the expected overall strength of insulation (datasets listed in Supplementary Data 1); (2) co-occupancy of subunits of the cohesin complex and the transcription factor Znf143^31^ from 38 published ChIP-seq datasets (Supplementary Data 1); (3) CTCF-binding conservation at orthologous regions in four different mammalian species^32^ (Supplementary Data 2); and (4) whether both flanking TADs contain genes with known roles in embryonic development, preferentially showing divergent patterns of tissue-specific expression (Fig. 1, Supplementary Fig. 1, Supplementary Data 1–3, “Methods”). TAD boundaries that encompassed protein-coding genes were excluded. Following genome-wide prioritization, we selected and deleted 8 individual TAD boundaries from the mouse genome through pronuclear injection of fertilized eggs using CRISPR/Cas9. For all deletions, the outer borders of the boundaries were defined by canonical criteria including the presence of CTCFs and cohesin complex proteins, which are the hallmark of TAD boundaries that are conserved across cell types in closely related species (refs. ^13,15–19^; see “Methods” for details). These deletions ranged in size from 11 to 80 kb and removed all known CTCF and cohesin binding sites in each TAD boundary region, while leaving any nearby protein-coding genes intact (Fig. 1, Supplementary Fig. 1, Supplementary Data 3, “Methods”). For all eight boundaries, live founder mice heterozygous for the targeted deletion were successfully obtained and bred into stable lines to assess molecular and organismal phenotypes.

### TAD boundary deletions disrupt prenatal development

**Fig. 2:**
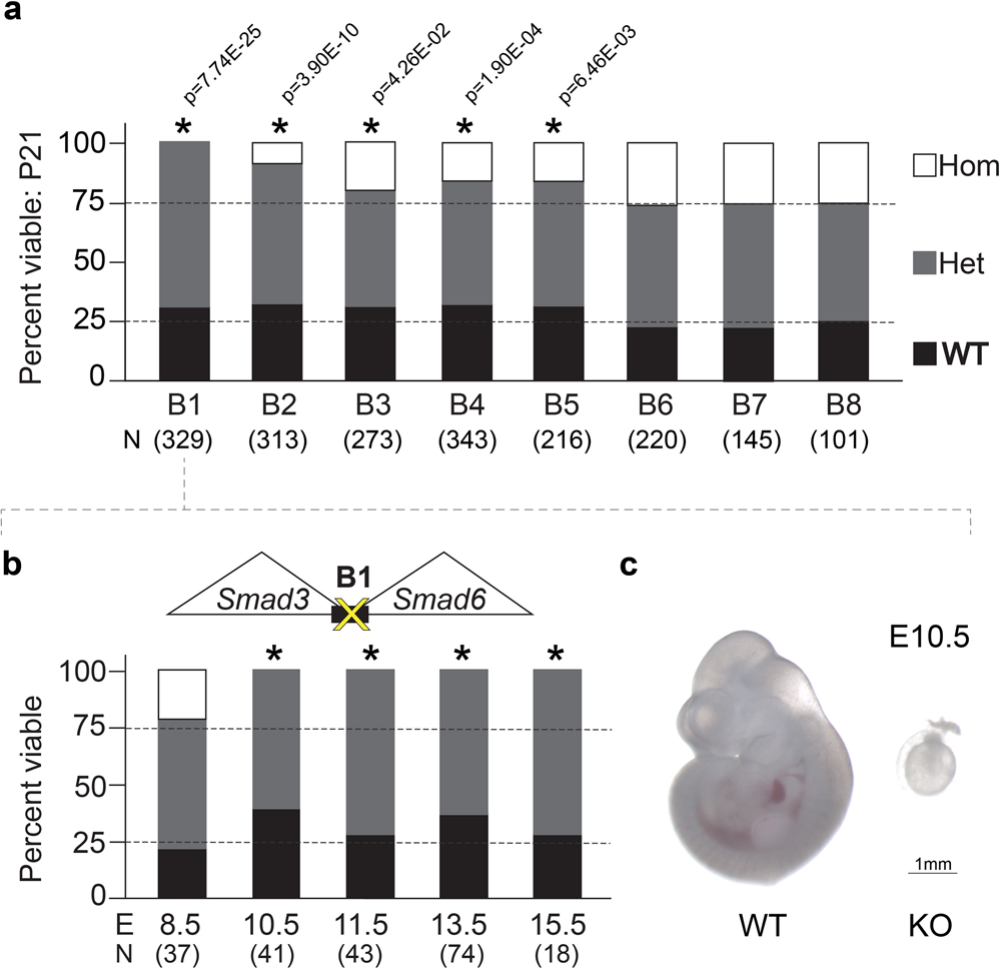
TAD boundary deletions result in reduced viability. **a** Mendelian segregation of offspring from heterozygous crosses at weaning for all TAD boundary deletion lines. P21 postnatal day 21, N number of pups analyzed, Hom homozygous, Het heterozygous, WT wild-type. **b** Mendelian segregation of offspring from heterozygous crosses at designated embryonic stages for boundary deletion locus B1 (*p < 0.05). E embryonic day, N number of embryos analyzed. **c** Brightfield images of representative littermate wild-type and homozygous mutant embryos obtained at E10.5. Scale bar, 1 mm.

To investigate the in vivo consequences of TAD boundary deletions, we assessed all lines for viability in homozygous offspring (Fig. 2a, Supplementary Data 4). First, we intercrossed heterozygous deletion mice to determine if homozygous offspring were viable and present at the Mendelian-expected rate (25%). For line B1, in which a boundary between *Smad3* and *Smad6* had been deleted, no mice homozygous for the deletion were observed among 329 live-born pups. Timed breeding revealed that homozygous embryos are present at embryonic day 8.5 (E8.5) at the expected Mendelian ratio but not at later stages of development (*p* < 0.05, Chi-squared test, for all examined stages E10.5 and later; Fig. 2b, and Supplementary Data 4). While no viable homozygous-null embryos were observed at E10.5, we observed partially resorbed homozygous deletion embryos at this stage, further corroborating that homozygous deletion of boundary B1 causes fully penetrant loss of viability between E8.5 and E10.5 (Fig. 2c, and “Methods”). Homozygous deletions of four additional boundary loci were associated with partially penetrant embryonic or perinatal lethality (Fig. 2a). For the most extreme (B2), we observed a loss of ~65% of expected homozygous offspring at weaning (*p* = 3.90E–10, Chi-squared test; Fig. 2a, Supplementary Fig. 2). For three additional boundary deletions (B3, B4, B5), we observed a 20–37% depletion of homozygotes at weaning (*p* < 0.05, Chi-squared test, in all cases, Fig. 2a, Supplementary Fig. 2). There were no significant sex biases among viable homozygous offspring in any of the lines (Supplementary Fig. 3, and Supplementary Data 4). Overall, these data show that the majority (5 of 8, 63%) of the boundary elements tested are required for normal organismal viability.

### TAD boundary deletions result in abnormal TAD architecture

**Fig. 3:**
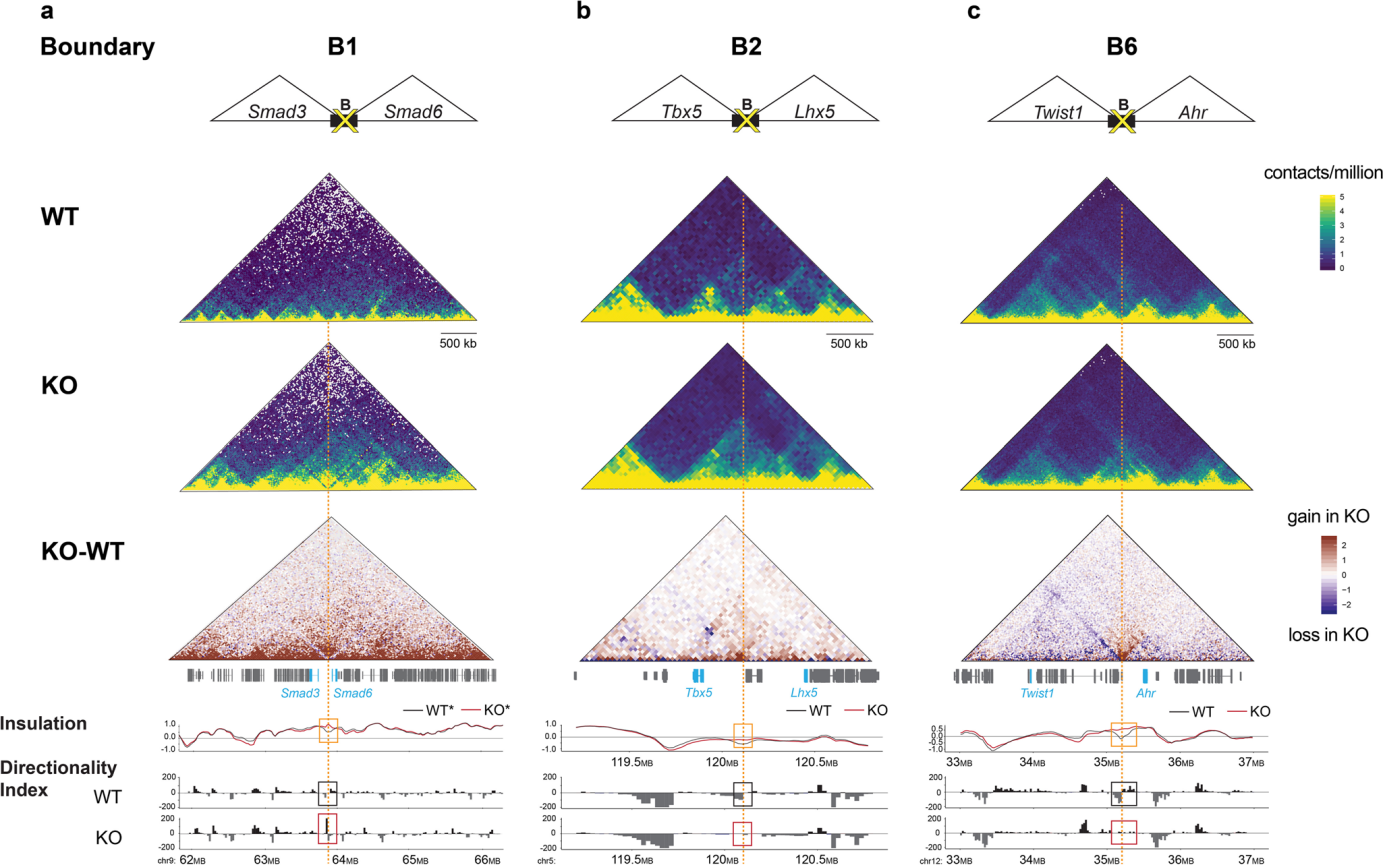
Boundary deletions result in abnormal TAD architecture. Hi-C derived interaction maps for TAD boundary loci B1 (**a**), B2 (**b**), and B6 (**c**). Cartoon of the TAD boundary deleted, with select developmental genes within the TADs flanking the deleted boundary (B), followed by three heatmaps showing Hi-C contact data. Heat maps (yellow-blue color-code) show Hi-C contact matrices presented as observed/expected contacts at 25 kb resolution in representative wild-type (WT) and knockout (KO) mouse liver tissue samples. For TAD boundary locus B1, note that the WT represents the wild-type allele in Cast background and KO represents the deletion allele in FVB background from an animal heterozygous for the TAD boundary deletion. The third heatmap (red-blue color-code) shows net changes in Hi-C interaction frequencies in the KO relative to WT. Positions of genes within the corresponding locus are indicated along the heatmap. The dashed orange vertical line indicates the position of the deleted boundary. Insulation profiles for the WT and KO samples corresponding to the heat maps are shown. The insulation profile assigns an insulation score to each genomic interval^50^, with local minima representing the most insulated region. Note the deviation from the minima in the insulation profile for the KO compared to WT (orange box), indicating loss of insulation in the KO. Bar plots show the Directionality Index (DI)^5^ for the same samples. Boundaries are called at regions where abrupt and significant shifts in upstream and downstream contacts are observed, as depicted in the WT (black box). Note either gain in contacts or the loss of demarcation between upstream and downstream contacts at the deleted site in the KO (red box). Genome coordinates shown are in mm10. More details on mouse strain background where applicable, replicates and additional TAD boundary deletions are provided in Supplementary Fig. 4 and “Methods”.

To assess the effects of TAD boundary deletions on the chromatin interaction landscape, we performed high-throughput chromosome conformation capture (i.e., Hi-C) using tissue from adult mice with homozygous deletions of TAD boundaries B2-B8 or heterozygous deletion of boundary B1, since homozygous B1 deletion is embryonically lethal (Fig. 3, Supplementary Fig. 4, and “Methods”). For four loci, we observed that homozygous-null mice displayed loss of insulation at the TAD boundaries and concurrent merging of neighboring TADs (loci B1– B3, and B6; Fig. 3, Supplementary Fig. 4). Loss of insulation was also observed at a fourth TAD boundary deletion (B8), but this was not associated with major changes to the overall TAD configuration at this locus (Supplementary Fig. 4). As a second measure of disrupted chromatin structure, we compared the directionality index (DI) between knockout mice and wild-type controls. DI assesses the trend of upstream (leftward or negative) or downstream (rightward or positive) contacts along a region of the chromosome^5^ and corner regions peripheral to TADs where abrupt shifts in upstream and downstream contacts are observed (i.e., sites with statistically significant contact biases are computationally called as boundaries between flanking TADs). Changes in DI were observed in homozygous-null mutants in 6 of the 8 lines assessed by Hi-C (*p* ≤ 0.01, Wilcoxon rank-sum test, for loci B1–4, B6, and B8; Fig. 3, Supplementary Fig. 4). Moreover, for three TAD boundary deletions (loci B4, B7, and B8) we observed a reduction of long-range contacts in one of the TADs adjacent to the deleted boundary (Supplementary Fig. 4). Taken together, we observed chromatin conformation changes in 88% (7 of 8) of lines examined. These data indicate that removal of individual TAD boundary sequences affects insulation between neighboring TADs, resulting in altered interaction frequency between sequences in normally isolated domains.

### TAD boundary deletions cause molecular and developmental phenotypes

We next examined whether loss of TAD boundaries and resulting changes in chromatin architecture were associated with additional molecular or physiological phenotypes. To determine if the deletions altered the expression of genes in the vicinity of each TAD boundary, we measured gene expression in E11.5 embryos with homozygous boundary deletions and matched wild-type controls. For each line, RNA-seq was performed in two different tissues with known expression of the genes located in the adjacent TADs, and qPCR was performed to query select genes in a larger panel of tissues for a subset of the TAD boundary deletion lines (Supplementary Fig. 5–6, and Supplementary Data 5–6). Across all seven lines examined by this approach, we identified two cases in which expression of a gene(s) in a TAD flanking the boundary changed. Embryos homozygous-null for B2 displayed a 44% reduction in *Tbx5* in the lungs as compared to WT controls (*p*_adj_ = 0.04, Supplementary Figs. 5–6, Supplementary Data 5–6). Downregulation of *Tbx5* in embryos homozygous-null for B2 was further corroborated by assessing *Tbx5* expression by in situ hybridization at stage E11.5 (Supplementary Fig. 7). Embryos homozygous-null for B6 showed ~40% reduction of expression of three genes (*Meox2*, *Sostdc1*, and *Prkar2b* in the heart; *Meox2* in the developing face; *p*_adj_ = 0.04, Supplementary Fig. 5 and Supplementary Data 5). While sampling only a subset of tissues at a single developmental timepoint, these data indicate that deletion of TAD boundaries alone can result in pronounced changes in tissue-specific gene expression.

**Fig. 4:**
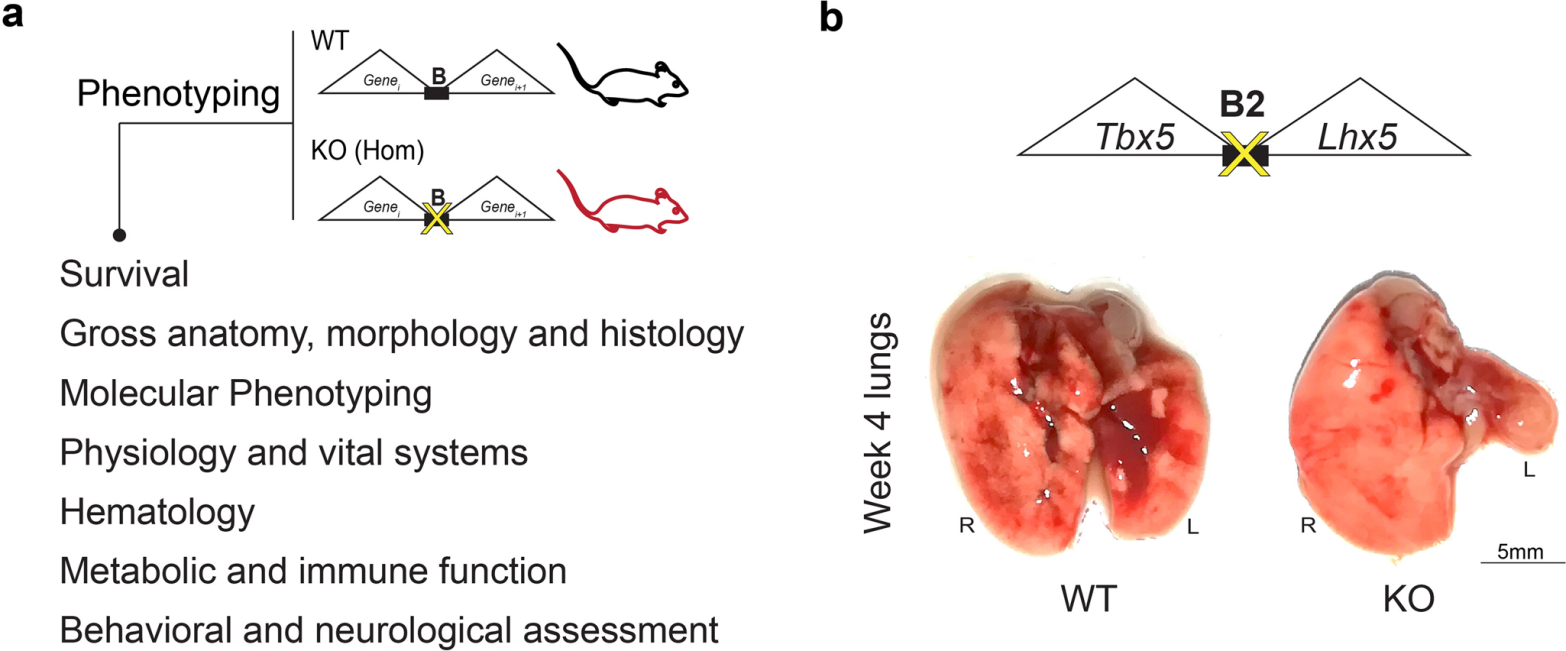
TAD boundary deletions result in developmental phenotypes. **a** Overview of comprehensive phenotypic assessment performed to characterize effects of TAD boundary deletion in vivo. **b** Homozygous mutants (KO) for TAD boundary deletion locus B2 show vestigial left lung. Scale bar, 5 mm.

Since most TAD boundary deletions did not result in fully penetrant embryonic lethality, we assessed postnatal phenotypes in lines with viable homozygous offspring (Fig. 4). In initial assessments of gross anatomy, morphology, and histology, the most remarkable phenotype observed occurred in mice homozygous for deletions of boundary B2, located between *Tbx5* and *Lhx5*, which show significantly reduced viability (Fig. 2a). Surviving knockout mice showed severely underdeveloped lungs, with a vestigial left lung (Fig. 4b). This phenotype is partially penetrant (12 out of 20 mice, or 60%) with higher rates observed in male homozygous mutants (82%) than females (33%). This lung anomaly is consistent with the downregulation of the nearby *Tbx5* gene in the developing lungs, as the phenotype has been observed in lung-specific knockouts of *Tbx5*^33^. Closer examination of the B2 boundary revealed that a subregion of the deleted region shows a lung-specific enhancer signature in the ENCODE epigenomic atlas of regulatory sequences^34,35^ (Supplementary Fig. 8a). We tested this 852 bp subregion region in a transgenic mouse reporter assay and observed that it was sufficient to drive reporter gene expression in the developing lungs across multiple mouse embryonic stages. (Supplementary Fig. 8b and “Methods”). However, neither targeted deletion of the enhancer sequence in isolation nor deletion of the enhancer in conjunction with an immediately adjacent CTCF binding site resulted in the lung phenotypes observed upon deletion of the full B2 boundary region (Supplementary Fig. 9), indicating that loss of the lung enhancer embedded in the boundary region is not the primary cause of the observed lung phenotype.

A subset of the remaining lines (B3, B5, B6, B7) was selected for comprehensive phenotyping based on parameters defined by the International Mouse Phenotyping Consortium (IMPC)^36–38^. The assays included a standardized panel of general anatomical, histological and necropsy examinations, including 230 sensory, neurological and behavioral tests, 20 tests measuring cardiac function, 35 metabolic function tests, 20 hematological/immunological parameters, and 36 musculo-skeletal tests, performed in 7–12 sex- and age-matched pairs of control and homozygous knockout mice from each line (>6000 data points across 350 total measurements; Fig. 4a, Supplementary Fig. 10 and Supplementary Data 7). These systematic phenotyping efforts identified 30 additional parameters with individually significant deviations from wild-type controls (Supplementary Data 7). However, due to the limited sample size and the large number of parameters assessed, these initial observations are generally not significant after correction for multiple hypothesis testing. Nevertheless, in conjunction with the independently observed viability, chromatin, and expression phenotypes in these lines, these data provide leads for further in-depth characterization.

## Discussion

**Fig. 5:**
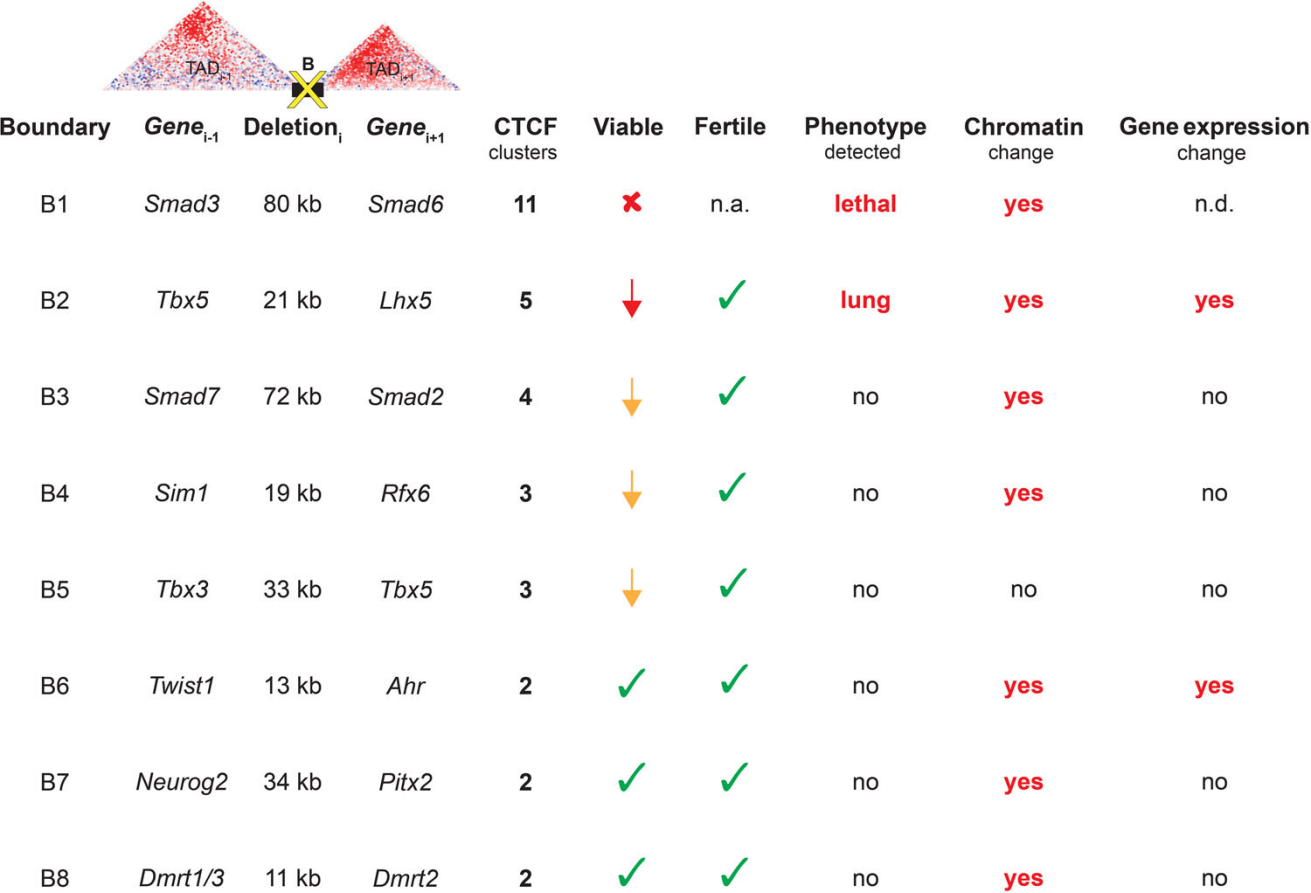
Summary of findings. Columns show specific TAD boundaries (B1–8) deleted in this study, key developmental genes within the flanking TADs (Gene_i-1_ and Gene_i+1_), approximate boundary deletion sizes (Deletion_i_), and the number of putative CTCF clusters deleted. All data in subsequent columns are summaries of phenotypic information for mice homozygous for the respective TAD boundary deletions. These include effects on viability (fully lethal in red cross mark, subviable shown in red or yellow arrow to indicate magnitude of effect, no deviation from expected Mendelian ratios shown in green check marks), fertility of both male and female homozygous animals (at least three homozygous males and three homozygous females were assessed for reproductive fitness, green check marks indicate fertile animals), and overt physiological phenotypes observed. Column for chromatin change indicates presence of significant DI changes and/or significant alterations to intra-domain chromatin contact frequencies in the flanking TADs, assessed by Hi-C. The last column indicates whether significant expression changes were observed for genes in the vicinity of the deleted TAD boundary. n.a. not applicable, n.d. not determined.

TAD boundaries have been hypothesized to be critical for normal genome function based on their known molecular roles in defining regulatory territories along chromosomes and in preventing enhancer-promoter interactions between adjacent chromatin domains. This notion was supported by observations of disease phenotypes associated with structural mutations that include TAD boundaries, although in most cases in combination with adjacent genomic features such as regulatory sequences or protein-coding genes^2,8,39,40^. To assess their general requirement for normal genome and organism function, we performed targeted genomic deletions of eight TAD boundaries in mice, using the canonical features of TAD boundaries based on molecular signatures (location between TADs; CTCF and cohesin binding sites) to select the end points of each deletion. We focused on genome regions that included genes with known developmental roles to facilitate the detection of possible phenotypes. Remarkably, all eight TAD boundary deletions resulted in abnormal molecular or organismal phenotypes (summarized in Fig. 5). This included seven lines showing alterations to chromatin interactions within or across neighboring TADs, two lines with substantial alterations of expression level of neighboring gene(s), five lines displaying complete or partial embryonic lethality, and one line showing defects in lung development.

Of the seven TAD boundary deletions that showed alterations in chromatin interactions, four resulted in merging of the neighboring TADs in the respective knockout lines (B1–3, B6), indicating severe disruption of boundary function. In the remaining cases, the adjacent TADs remained overall intact despite having deleted the TAD boundary region as defined by canonical TAD features. This implies that current approaches for defining the outer borders of domain boundary elements may be imperfect and that additional local determinants may play instructive roles in chromatin domain formation and maintenance. Intriguingly, we observed reduced viability in two lines (B4, B5) in the absence of merged TADs, suggesting functional impacts of boundary deletions that are independent from the merging of neighboring domains. This is well aligned with the notion that even subtle changes in three-dimensional chromatin structure may result in substantial changes in local gene expression^41^. Our results point to regulatory or other functional impacts of boundary deletions that occur in the absence of the complete merging of neighboring domains. For example, the loss of boundary B4 does not result in domain merging but does lead to decreased organismal viability, which presumably results from subtle changes in chromatin structure and downstream regulatory mechanisms.

Concomitant to the changes in chromatin conformation, we observe changes in gene expression for specific tissues at the time point examined for two (B2 and B6) of seven TAD boundary loci. For deletion B6, genes with altered expression (*Meox2*, *Sostdc1*, *Prkar2b*) are in regions without significant changes in the three-dimensional context as compared to the wild-type configuration. One possible explanation is that more subtle changes to subtopologies nested within flanking TADs were not detected at the resolution of our conformation data, but are sufficient to disrupt interactions between regulatory elements and promotors of dosage-sensitive genes^42,43^.

For mice with a homozygous deletion of boundary B1, fully penetrant embryonic lethality was observed. While the exact developmental process affected was not assessed in this study, this result demonstrates a critical role of this boundary for viability and normal development. Boundary B2 deletion caused a pronounced lung malformation that recapitulates a phenotype associated with the lung-specific deletion of the nearby *Tbx5* gene^33^. Intriguingly, this boundary contains a developmental lung enhancer in the immediate vicinity of a major CTCF binding site but neither deletion of the enhancer, nor deletion of the CTCF site, nor deletion of these two small subregions in combination recapitulates the phenotype observed upon deletion of the complete B2 boundary region. This observation reinforces the possibility that critical molecular functions are embedded across the length of the extended TAD boundary region, which may include the presence of additional, functionally redundant lung enhancer elements^44^ that are affected by the B2 deletion. Our findings highlight the need for a better understanding of the spatial delimitations of boundary regions within the genome. For TAD boundary loci where we observe deviations from normal viability but identified no other obvious physiological phenotypes (B3–5), the molecular mechanisms underlying the survival phenotypes remain to be established. While we performed a reasonably comprehensive survey to assess possible gene expression and organismal phenotypes, we cannot exclude the presence of additional gene expression or other phenotypic changes that manifest at developmental time point or in a tissue or environmental condition other than those examined. We also cannot exclude the presence of very subtle phenotypes, for example changes in developmental timing.

The boundary deletions performed in this study caused a spectrum of phenotypes, ranging from severe (complete embryonic lethality) to mild (molecular phenotypes only). Likewise, the chosen boundaries ranged widely in the number of CTCF clusters present, from two in boundaries that caused altered chromatin interactions or gene expression changes only, to three to five in the boundaries that cause reduced viability, to 11 CTCF clusters in the boundary that caused the most severe phenotype. The dosage of CTCF at TAD boundaries is known to affect the formation of TADs^22,45,46^, and while the number of boundaries studied here is too small to establish a statistically robust correlation, it is tempting to speculate that deletions of boundaries with more CTCF sites tend to cause more pronounced phenotypes.

Our findings have important implications for interpreting human whole genome sequencing data in clinical genetic settings. Position effects ensuing from large structural variations are well known in human genetics^47^ but it is often difficult to disconnect such deletions and/or rearrangements of functional sequences, such as protein-coding genes and enhancers, from effects that are due to removal of boundary sequences. Remarkably, none of the eight regions deleted from the mouse genome in this study is completely deleted in ~760,000 available human genomes, consistent with their functional importance (gnomAD-SV v2.1^48^ and rCNV2^49^). A critical future goal will be to determine the relationship between human mutations within the thousands of genome-wide TAD boundaries and their impact on human phenotypic variation and disease. The present study indicates that removal of the relatively small TAD boundary sequences themselves causes molecular or organismal phenotypes and, therefore, structural variants that include TAD boundary deletions in human patients should be considered as potential causes of pathogenicity.

## Methods

### ENCODE ChIP-seq data analysis and prioritization of TAD boundary deletion loci

Previously published chromosome conformation capture data was used for combined analyses and selection of TAD boundary deletion loci in this study, wherein TAD boundary calls were based on the maximum enrichment of CTCF at the TAD borders and their consistency across cell-types^10^. Over 3300 genome-wide annotated TAD boundaries^5,10^ were then ranked on a weighted score (see Supplementary Fig. 1), encompassing strength of insulation based on CCCTC-binding factor (CTCF) occupancy, co-occupancy of subunits of the cohesin complex and the transcription factor Znf143^31^ and CTCF-binding conservation at orthologous regions in four different mammalian species^32^ (Fig. 1, Supplementary Fig. 1). We analyzed ~100 individual ChIP-seq datasets to this effect (listed in Supplementary Data 1). CTCF-bound sites genome-wide were spatially clustered and individually scored based on the criteria highlighted above within regions 40 kb upstream and downstream of each TAD boundary. An overall score for each boundary was devised based on the intervening bound CTCFs, excluding boundaries overlapping protein-coding genes, thus enabling unambiguous interpretation of the functional necessity of TAD boundaries in mammalian development. Furthermore, boundaries where flanking TADs harbored genes encoding transcription factors important for development and preferentially (to the extent possible) showing a divergent pattern of tissue-specific expression were prioritized for in vivo deletion. Our selection criteria did not factor in the directionality of CTCF motifs when selecting TAD boundary loci for deletion (Supplementary Fig. 1).

### Experimental design of mouse studies

All animal work was reviewed and approved by the Lawrence Berkeley National Laboratory Animal Welfare Committee. Mice were monitored daily for food and water intake, and animals were inspected weekly by the Chair of the Animal Welfare and Research Committee and the head of the animal facility in consultation with the veterinary staff. The LBNL ACF is accredited by the American Association for the Accreditation of Laboratory Animal Care (AAALAC). TAD boundary knockouts were performed in *Mus musculus* FVB strain mice with an exception for TAD boundary B1 where heterozygous-null mice in a mixed strain background (Cast/Eij and FVB) were necessary to be generated specifically for conducting the Hi-C assays. Mice across developmental stages from embryonic day 10.5 through P0, as well as mice between weeks 4–16 were used in this study. Animals of both sexes were used in the analysis. Sample size selection and randomization strategies are included in individual method sections. Unless otherwise stated, all phenotyped mice described in the paper resulted from crossing heterozygous TAD boundary deletion mice together to allow for the comparison of matched littermates of different genotypes. Samples were dissected and processed blind to genotype where applicable.

### Generation of TAD boundary deletion mice, and specific regulatory sequence deletions for TAD boundary B2

Transgenic mice were generated using the *Mus musculus* FVB strain and a standard CRISPR/Cas9 microinjection protocol^50^. Briefly, Cas9 protein (Integrated DNA Technologies catalog no. 1081058) at a final concentration of 20 ng/μl was mixed with sgRNA targeting the intended locus (50 ng/μl, for all sgRNAs combined), in microinjection buffer (10 mM Tris, pH 7.5; 0.1 mM EDTA). The mix was injected into the pronuclei of single cell stage fertilized FVB embryos obtained from the oviducts of super-ovulated 7–8 weeks old FVB females mated to FVB males (See Supplementary Data 8 for sgRNA sequences). The injected embryos were then cultured in M16 medium supplemented with amino acids at 37 °C under 5% CO_2_ for ~2 h. The embryos were subsequently transferred into the uteri of pseudo-pregnant CD-1 surrogate mothers. Founder (F0) mice were genotyped using PCR with High Fidelity Platinum Taq Polymerase (Thermo Fisher) to identify those with the desired NHEJ-generated deletion breakpoints. Sanger sequencing was used to identify and confirm deletion breakpoints in F0 and F1 mice (Supplementary Fig. 11, Supplementary Data 8 for CRISPR sgRNA templates and Supplementary Data 9 for primer sequences and PCR amplicons). Between one and four F0 founders were obtained for each of the TAD boundary deletion loci, each of which were simultaneously assayed for possible inversions by PCR. Only those F0 founders that harbored clean deletion alleles were backcrossed to wild-type mice and bred to procure F1 heterozygous mice. Given that each of the deletions across founders were consistent in the NHEJ-mediated deletion span, only one founder line for each locus was eventually selected to expand breeding for experiments in this paper. Additional confirmation and visualization of the deleted TAD boundaries is evident in Hi-C contact matrices resulting from Hi-C experiments on tissue from homozygous TAD boundary mutants compared to wild-type mice (Supplementary Fig. 11).

Generation of deletion mice for selected regulatory sequences within TAD boundary B2 encompassed individual deletions of a lung enhancer (enhancer Δ), an adjacent CTCF site (CTCF Δ) and deletion of the enhancer along with the CTCF site (enhancer+CTCF Δ) as control. These mice were generated identical to the methods described above and details are provided accordingly (Supplementary Fig. 12, Supplementary Data 8 for CRISPR sgRNA templates and Supplementary Data 9 for primer sequences and PCR amplicons).

In addition, mice heterozygous for TAD boundary B1 were used for conducting Hi-C assays for this TAD boundary knockout line. However, to circumvent the problem of distinguishing the alleles bioinformatically, mice in a mixed stain background were necessary to assess the changes in Hi-C contacts upon boundary deletion. To this end, we obtained wild-type Cast/Eij mice from the Jackson Laboratory and crossed these with our existing FVB mice that were heterozygous for the TAD boundary deletion B1. The heterozygous mice resulting from these crosses resulted in TAD boundary B1 heterozygous animals that harbored Cast/Eij background for the wild-type allele and concomitant FVB background for the deletion allele.

The described mouse lines are made available through the Mutant Mouse Resource and Research Center, www.mmrrc.org, and can be found in the MMRRC catalog using the regulatory region symbol, or the Research Resource Identifiers (RRID) (Supplementary Data 10).

### Assessment of Mendelian segregation and viability

Sample sizes were selected empirically based on our previous studies^44,51^. Mendelian segregation was initially assessed postnatally on animals resulting from heterozygous crosses, thus allowing for comparison of matched littermates of different genotypes. Where applicable, Mendelian ratios were assessed in embryological time points as necessitated by the phenotype on a case-by-case basis. For TAD boundary B1, although we have rarely obtained homozygous-null mutants at E13.5, no viable homozygous-null embryos were observed by E10.5 in a systematic assessment of Mendelian segregation in  213 embryonic samples harvested between embryonic days 8.5-15.5 for this knockout line (Supplementary Data 4).

### In situ Hi-C library generation

Hi-C experiments were performed on ex vivo liver tissue from male mice at post-natal day 56. Upon euthanasia, liver samples were harvested, flash frozen in liquid nitrogen and pulverized before 1% formaldehyde cross-linking for 15 min. Thawed crosslinked tissue was dissociated by a gentleMACS Tissue Dissociator using the factory-set program and filtered through a 40 µm BD-cellstrainer. Cell pellets were centrifuged at 1000 × *g* for 6 min at 4 °C and overlaid with 3 ml 1 M sucrose. The suspension was centrifuged at 2500 × *g* for 6 min at 4 °C. Pelleted nuclei were resuspended in 50 μL 0.5% SDS and incubated for 10 min at 62 °C. SDS was quenched by adding Triton X-100 and incubation for 15 min at 37 °C. Chromatin was digested using MboI (100U; NEB) at 37 °C overnight with shaking (1000 rpm). The enzyme was inactivated by heating 20 min at 62 °C. Fragmented ends were labeled with biotin-14-dATP (Life Technologies) using Klenow DNA polymerase (0.8 U μl^−1^; NEB) for 60 min at 37 °C with rotation (900 rpm). Ends were subsequently ligated for 4 h at room temperature using T4 DNA Ligase (4000 units; NEB). Reverse crosslinking was performed using Proteinase K (1 mg, NEB) and incubation at 55 °C overnight. The digestion efficiency and ligation efficiency were checked by gel electrophoresis. Next, DNA was purified by using ethanol precipitation and sheared using a Covaris Focused-ultrasonicator (M220; duty cycle: 10%; Power: 50, Cycles/burst: 200, Time: 70 s). After size selection and purification using SPRI beads (Beckman Coulter), DNA was biotin pulled-down using Dynabeads MyOne Streptavidin T1 beads (Life Technologies). Finally, sequencing libraries were prepared on T1 magnetic beads, and final PCR amplification was performed for seven cycles based on qPCR analysis. Bead-purified libraries were quantified with a Qubit and then diluted for size distribution assessment using High Sensitivity D1000 ScreenTape on a TapeStation (Agilent).

Hi-C was performed on two biological replicates each for both homozygous-null and control samples for each of the TAD boundary deletion loci B2–8. For TAD boundary B1, Hi-C was performed on two biological replicates that were heterozygous-null for the boundary deletion and one wild-type sample, and all these samples were generated from mixed-strain (Cast/FVB) background mice for the following reasons (i) the early embryonic lethality of homozygous mutants for this mouse line, (ii) the requirement of large amount of tissue for performing Hi-C experiments, (iii) limitations of using heterozygous mutants in isogenic FVB background for Hi-C experiments, as these would make allele-specific downstream analyses problematic. Details on generating these mice are described in “Methods”.

### Hi-C data analysis

The Hi-C data processing pipeline is available at https://github.com/ren-lab/hic-pipeline. Briefly, with respect to TAD boundary loci B2–8, Hi-C reads were aligned to the mouse mm10 reference genome using BWA-MEM^52^ for each read separately, and then paired. For TAD boundary locus B1, where tissue from animals in a mixed strain background were used, we constructed both Cast and FVB genome sequences using the SNP information, then used BWA-MEM^46^ to align the raw reads to both Cast and FVB genome sequence; next, for each read, we compared the two mapping qualities and the mapped length, we chose the mapping results with higher scores. Beyond this analyses step, downstream analyses for all TAD boundary loci followed the same standards. For chimeric reads, only 5′ end-mapped locations were kept. Duplicated read pairs mapped to the same location were removed to leave only one unique read pair (MarkDuplicates in Picard package). The output bam files were transformed into juicer file format for visualization in Juicebox^53,54^. Contact matrices were presented as observed/expected contacts at 25-kb resolution and normalized using the Knight–Ruiz matrix balancing method^55^. Directionality index for each sample was also generated at 25-kb resolution^5^. Insulation score for each sample was generated at 25-kb resolution with 500-kb square^56^. For data in Fig. 3, Directionality Index (DI) scores of five bins on the right and five bins on the left were averaged, prior to calculating the difference. A higher DI delta score indicates a stronger boundary. A Wilcoxon rank-sum test was performed between KO and WT samples using DI delta scores, and a *p*-value ≤ 0.05 considered significant. As a negative control, the same statistical test was performed on ~2900 TAD boundaries that do not overlap with deletions, and differences between WT and KO samples were assessed by the same statistical test, using a significance threshold of *p* ≤ 0.05.

We did not observe any other boundaries genome-wide that showed a significant difference in DI delta score that exceeded that of deleted boundaries B2, B3, B4, B6, and B8, for which we had observed significant changes in DI delta score. Twelve boundaries (0.4%) genome-wide showed changes that were significant (*p* ≤ 0.05) and were quantitatively the same or exceeded those observed at deleted TAD boundaries B5 and B7, for which we did not observe significant changes in DI delta score (*p* > 0.05; Fig. 3 and Supplementary Fig. 4. Interaction frequencies between genomic loci were additionally visualized on Juicebox (Supplementary Fig. 4)^53,54^.

### RNA-seq and quantitative real time PCR

A panel of tissues including forebrain, midbrain, hindbrain, face, heart, upper and lower limbs and neural tube was collected in a standardized manner at E11.5 from homozygous mutants as well as littermate wild-type embryos for each of the TAD boundary deletion loci^34^. Samples were suspended in 100 μl of commercially available (Qiagen) RLT buffer. Total RNAs were isolated by using the Qiagen RNeasy Mini Kit (catalog no. 74104). A set of two relevant tissue types was further selected for each of the TAD Boundary deletion loci B2-B8 and processed for RNA-seq libraries in a standardized manner. Sequencing libraries were prepared by Novogene, and sequenced on an Illumina NovaSeq6000 (150 bp, paired-end). RNA-seq data was analyzed using the ENCODE Uniform Processing Pipelines (https://www.encodeproject.org/pipelines/) implemented at DNAnexus (https://www.dnanexus.com). Using the ENCODE RNA-seq (Long) Pipeline – 1 (single-end) replicate pipeline (code available from https://github.com/ENCODE-DCC/rna-seq-pipeline), reads were mapped to the mouse genome (mm10) using STAR align (V2.12). Genome-wide coverage plots were generated using bam to signals (v2.2.1). Gene expression counts were generated for gencode M4 gene annotations using RSEM (v1.4.1). Differential expression analyses were performed by using the DESeq program in the R Statistical Package https://bioconductor.org/packages/3.3/bioc/vignettes/DESeq/inst/doc/DESeq.pdf^57,58^. Statistically significant differentially expressed genes for relevant tissues for each TAD boundary deletion locus are listed in Supplementary Fig. 5, and Supplementary Data 5. RNA-seq experiments were performed on two biological replicates each for homozygous mutants, as well as wild-type controls.

Quantitative PCR analysis of key developmental genes in the vicinity of each TAD boundary in a larger panel of E11.5 tissues did not identify any additional significant changes in gene expression (Supplementary Fig. 6, and Supplementary Data 6). For the comprehensive panel of tissues collected, RNA was isolated as described above and cDNA was synthesized using Omniscript RT (Qiagen catalog no. 205111) per standard methods. qPCR assays were performed for at least two genes, each in TADs immediately flanking the deleted boundary. Taqman Assay reagents (Life Technologies) were used for all targets including genes that were used to normalize expression levels. Taqman assays (Roche Applied Science) with gene-specific primer sequences were generated using the manufacturer’s online algorithm and are listed in Supplementary Data 11. All amplicons span exon-exon junctions to prevent amplification of genomic DNA. 30 μl assays dispensed in TaqMan Universal PCR 2X master mix (Applied Biosystems) were performed on LightCycler 480 (Roche) according to manufacturer’s instructions. All Ct values were manually checked. Relative gene expression levels were calculated using the 2^−ΔΔCT^ method^59^ normalized to the *Actb* housekeeping gene, and the mean of wild-type control samples was set to 1. At least three independent mutant samples and littermate and stage matched controls were assessed for each genotype/condition. We did not observe significant expression changes near deleted boundaries B3, B5 and B7. Considering RNA-seq analysis was performed on bulk tissue from a single developmental timepoint, we cannot exclude that expression changes are restricted to subsets of cells present in these tissues or may be more pronounced at other developmental timepoints.

### In situ hybridization assay

To assess the expression of *Tbx5* in developing lungs for control and TAD-boundary deletion mutants for locus B2, whole-mount in situ hybridization using digoxigenin-labeled anti-sense RNA probes was carried out on E11.5 (48 somites-stage) mouse embryos following established protocols^60,61^. Samples were treated with Proteinase K for 15 min; embryos were dissected to remove the hearts to expose the lungs and subsequently imaged with a Leica 125 C stereomicroscope with a Flexacam C1 camera (Supplementary Fig. 7).

### Comprehensive mouse phenotyping

Directly relevant results are summarized in Fig. 4. Mutants and wild-type controls for four out of eight TAD boundary deletion loci (B3, B5, B6 and B7) underwent comprehensive phenotyping using a standardized pipeline at the Mouse Biology Program (MBP), University of California, Davis. The pipeline is part of the NIH-funded Knockout Mouse Phenotyping Project (KOMP2), a participant in the International Mouse Phenotyping Consortium (IMPC). Phenotyping tests are derived from the International Mouse Phenotyping Resource of Standardized Screens (IMPReSS), https://www.mousephenotype.org/impress^36^, all protocols and metadata are accessible at https://www.mousephenotype.org/impress/PipelineInfo. The KOMP2 phenotyping (Supplementary Figure 10) and statistical analysis methods are standardized^37,38^. Supplementary Data 7 summarizes other statistically significant (*p* < 0.005) results reported. Mutants and wild-type controls for the other three TAD boundary deletion loci (B2, B4 and B8) were phenotyped using standard methods for gross necropsy, organ weights and histopathology for all major organ systems at the Comparative Pathology Laboratory, University of California, Davis.

### In vivo transgenic enhancer-reporter assay

Transgenic enhancer-reporter assays for the predicted lung enhancer (852 bp) were performed in a site-directed transgenic mouse assay using a minimal *Shh* promoter and *lacZ* reporter gene (Supplementary Fig. 8) at a non-endogenous, safe harbor locus^50^. The predicted enhancer region was PCR amplified from mouse genomic DNA**;** chr5:120101603–120102454 (mm10), CTGGGCTACAGGAAGTTGGA (forward primer), CAGAGGGCATGAGAGAGACC (reverse primer), 852 bp PCR amplicon. The PCR amplicon was cloned into a *lacZ* reporter vector (Addgene #139098) using Gibson assembly (New England Biolabs)^62^. The final transgenic vector consists of the predicted enhancer–promoter–reporter sequence flanked by homology arms intended for site-specific integration into the *H11* locus in the mouse genome^50^. Sequence of the cloned constructs was confirmed with Sanger sequencing as well as MiSeq. Transgenic mice were generated using pronuclear injection, as described above for generating the TAD boundary deletion mice. F0 embryos were collected for staining at E11.5, E14.5 and E16.5.

β-galactosidase staining was performed in a standardized manner^50^. Briefly, embryos were washed in cold 1× phosphate-buffered saline (PBS) and fixed with 4% paraformaldehyde (PFA) for 30 min for E11.5 and 60 min for E14.5 and E16.5 embryos, respectively, while rolling at room temperature. The embryos were washed in embryo wash buffer (2 mM magnesium chloride [Ambion, catalog no. AM9530], 0.02% NP-40 substitute [Fluka, catalog no. 74385], 0.01% deoxycholate [Sigma-Aldrich, catalog no. D6750] diluted in 0.1 M phosphate buffer, pH 7.3) three times for 30 min each at room temperature and transferred into freshly made X-gal staining solution (4 mM potassium ferricyanide [Sigma-Aldrich, catalog no. P3667], 4 mM potassium ferrocyanide [Sigma-Aldrich, catalog no. P9387], 20 mM Tris, pH 7.5 [Invitrogen, catalog no. 15567027], 1 mg ml^−1^ of X-gal [Sigma-Aldrich, catalog no. B4252]). Embryos were incubated in the staining solution overnight while rolling at room temperature and protected from light. Embryos were then washed with 1× PBS three times for 30–60 min per wash and subsequently stored in 4% PFA at 4 °C. The embryos were genotyped for presence of the transgenic construct^50^. Only those embryos positive for transgene integration into the *H11* locus and at the correct developmental stage were considered for comparative reporter gene activity across the three constructs tested. The exact number of embryos are reported in Supplementary Fig. 8.

### Statistics and reproducibility

Statistical analyses are described in detail in the Methods. For Mendelian ratios, between 18–43 embryonic samples (TAD boundary locus B1) and between 101–343 (B1–8) mice at weaning stage were systematically collected, and Chi-squared test was used to determine significant deviations. At least two independent biological samples per condition were analyzed for RNA-seq, and at least three independent biological samples per condition were analyzed for qPCR at e11.5; a Likelihood-ratio test (DESeq) and t-test were used to determine significant differences for RNA-seq, and qPCR results respectively. At least two independent biological samples per condition were analyzed for Hi-C experiments, and a Wilcoxon rank-sum test was used to calculate *p*-values. Between 7 and 12 independent post-natal mice per condition were assessed in the standardized IMPC pipeline, and either a Wilcoxon rank-sum test, Fisher’s exact test or PhenStat Mixed Model or Reference Range tests were used as applicable. For TAD boundary B2, 20 stage- and litter-matched homozygous mutant-control pairs were assessed for gross lung morphology. Between 4–9 independent biological samples were assessed for the in vivo transgenic reporter assays showing enhancer activity in embryonic lungs. For the enhancer-, CTCF-, and enhancer+CTCF-specific deletions in the context of TAD boundary B2, between 3–8 independent mice per genotype were assessed for gross lung morphology. All statistics were estimated, and plots were generated using the statistical computing environment R Version 2022.12.0 + 353 (www.r-project.org).

### Reporting summary

Further information on research design is available in the Nature Portfolio Reporting Summary linked to this article.

## Supplementary information

Supplementary Information

Description Of Additional Supplementary Files

Supplementary Data 1

Supplementary Data 2

Supplementary Data 3

Supplementary Data 4

Supplementary Data 5

Supplementary Data 6

Supplementary Data 7

Supplementary Data 8

Supplementary Data 9

Supplementary Data 10

Supplementary Data 11

Reporting Summary

## Data Availability

The Hi-C and RNA-seq data discussed in this publication have been deposited in NCBI’s Gene Expression Omnibus^63,64^ and are accessible through GEO Series accession number GSE172089. The source data for Fig. 2 and Supplementary Fig. 3 are provided in Supplementary Data 4, those for Supplementary Fig. 5 are provided in Supplementary Data 5, and those for Supplementary Figure 6 are provided in Supplementary Data 6. The source data for Supplementary Figures 11–12 are provided in Supplementary Figures 13–14 respectively. Additional data supporting the findings of this study are available from the corresponding authors upon reasonable request.

## Diversity and inclusion statement

We worked to ensure sex balance in the selection of non-human subjects. We worked to ensure diversity in experimental samples through the selection of the genomic datasets. One or more of the authors of this paper self-identifies as an underrepresented ethnic minority in science. One or more of the authors of this paper received support from a program designed to increase minority representation in science. While citing references scientifically relevant for this work, we also actively worked to promote gender balance in our reference list. The author list of this paper includes contributors from the location where the research was conducted who participated in the data collection, design, analysis, and interpretation of the work.
