# Supplementary material for: Topologically associating domain boundaries are required for normal genome function
Source: Commun Biol. 2023 Apr 20;6:435. doi: 10.1038/s42003-023-04819-w (PMC10119121; doi:10.1038/s42003-023-04819-w)
Supplement: Supplementary file 1 — Supplementary Information [file 42003_2023_4819_MOESM1_ESM.pdf]

## Supplementary Information

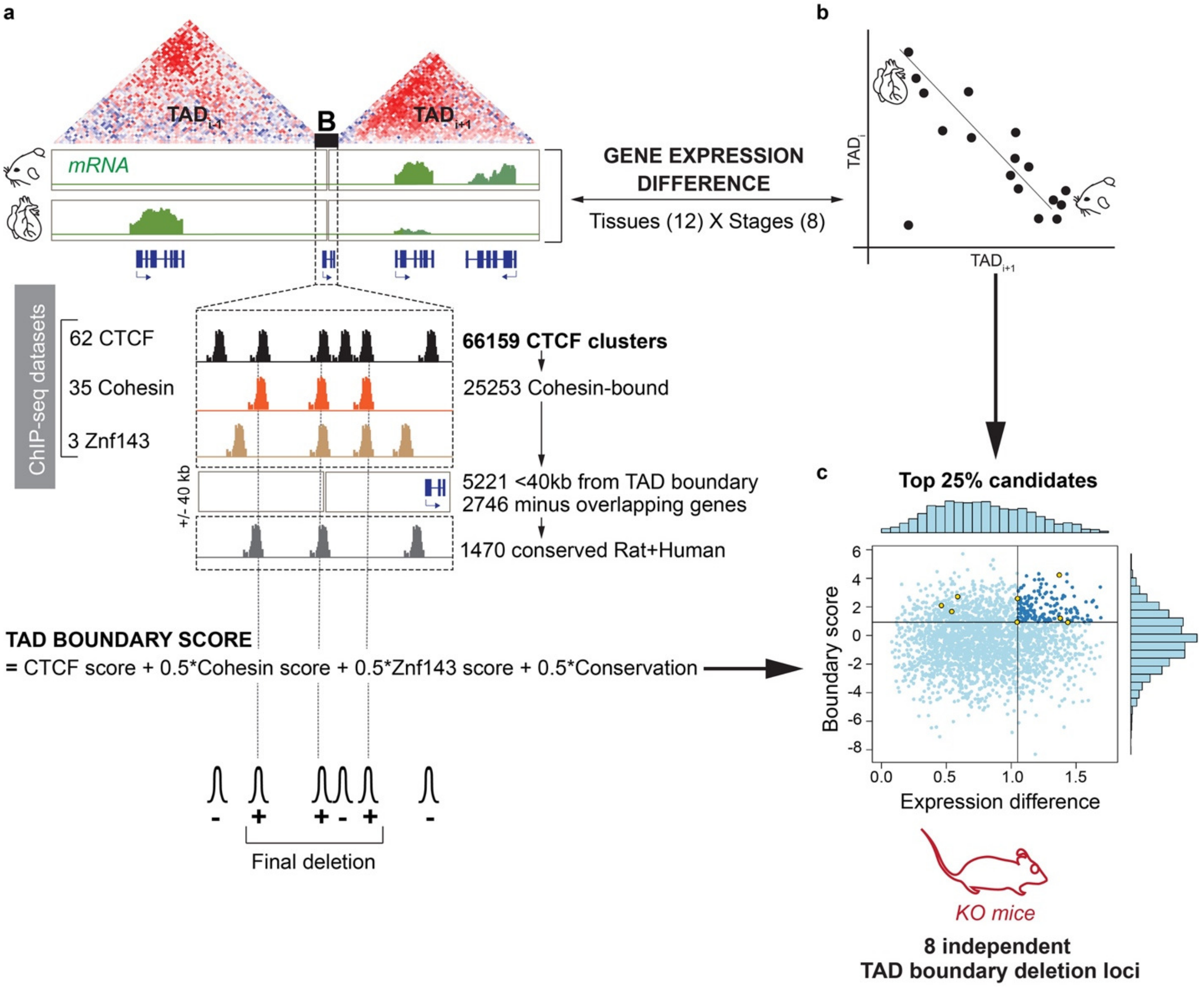


**Supplemental Figure 1. Schematic describing details of TAD boundary score and gene expression differences for selecting TAD boundary loci for deletion. a.** CTCF clusters at TAD boundaries were systematically scored for co-bound Cohesin and Znf143. Conservation of CTCF sites between rodent and human was also factored into the weighted score. **b.** We used an extensive collection of gene expression data from embryonic mouse tissues to assess differences and similarities between developmental genes on opposite sides of each boundary[1]. The rationale for selecting TAD pairs harboring genes with divergent expression profiles was to enable straightforward scoring of molecular phenotypes that are expected to result from TAD boundary disruption and perturbation of typical contacts between *cis*-regulatory elements and their cognate genes. **c.** We selected 8 TAD boundaries for deletion (yellow points) from the top 25% of candidates based on high boundary score, as well as moderate to high gene expression differences for genes in neighboring TADs.

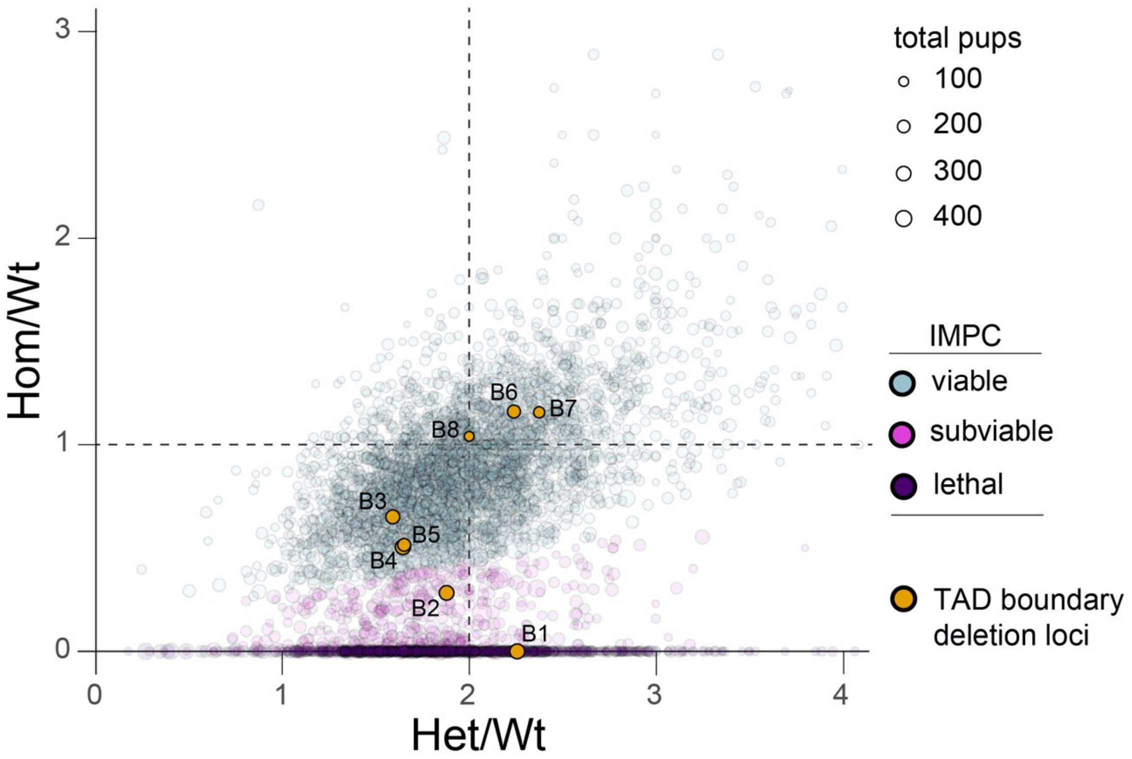


**Supplemental Figure 2. Allelic ratios of TAD boundary deletion KO lines compared with International Mouse Phenotyping Consortium (IMPC) mouse viability data**. This comparison is based on data analyses from Cacheiro *et al*, Nat. Commun., 2020[2] and Mendelian segregation data publicly available for homozygous null mice generated by IMPC. This plot shows viability data by genotype ratios before weaning stages for independent KO lines corresponding to 4866 mouse genes from the IMPC curated data. Sizes of circles correspond to sample size of total pups genotyped for a given gene KO mouse line. IMPC defines categories as follows: KO lines are defined as “Viable” (gray) when homozygous, heterozygous and wild-type pups for a given gene KO are obtained at expected frequencies; KO lines where approximately half of the expected number of homozygotes (δ 12.5%) are obtained are defined as “subviable” (magenta); and KO lines where no homozygotes are obtained are defined as “lethal” (violet). The TAD boundary deletion lines in this study are shown in orange. No homozygotes were obtained at birth for locus B1, and boundary deletions for loci B2 and B3, respectively, resulted in approximately 65% and 20% fewer homozygous pups. B4 and B5 resulted in approximately 37% fewer homozygous pups at weaning. While this strongly suggests partially penetrant embryonic lethality in TAD boundary KO lines B3-5, deviations from the expected offspring ratios are within the range for lines commonly reported as viable in IMPC gene knockout analyses. See **Figure 2** and **Methods** for details.

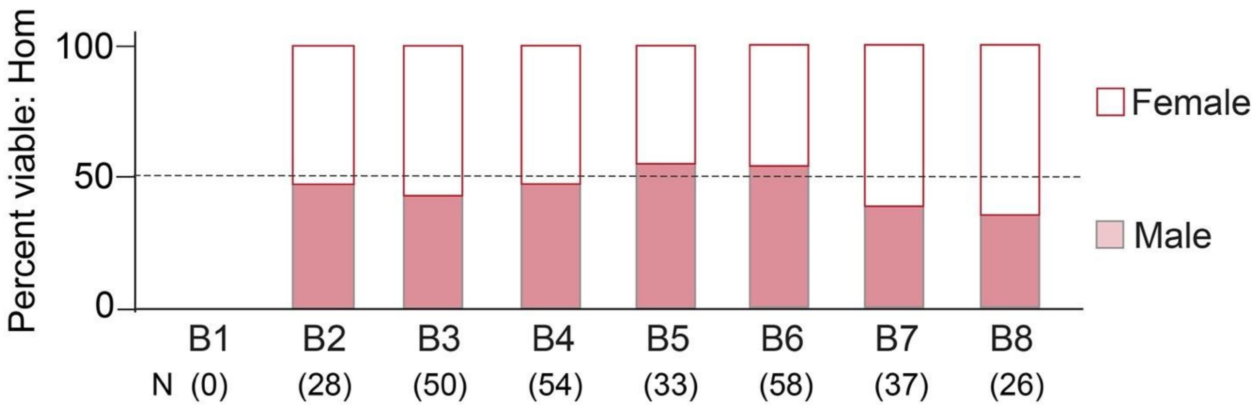


**Supplemental Figure 3. TAD boundary deletions show no sex bias within homozygous mutants at weaning.** Mendelian segregation of male and female homozygous offspring from heterozygous crosses at weaning (approximately postnatal day 20, or P20) for all TAD boundary deletion lines. See **Supplementary Data 4** and **Methods** for additional details.

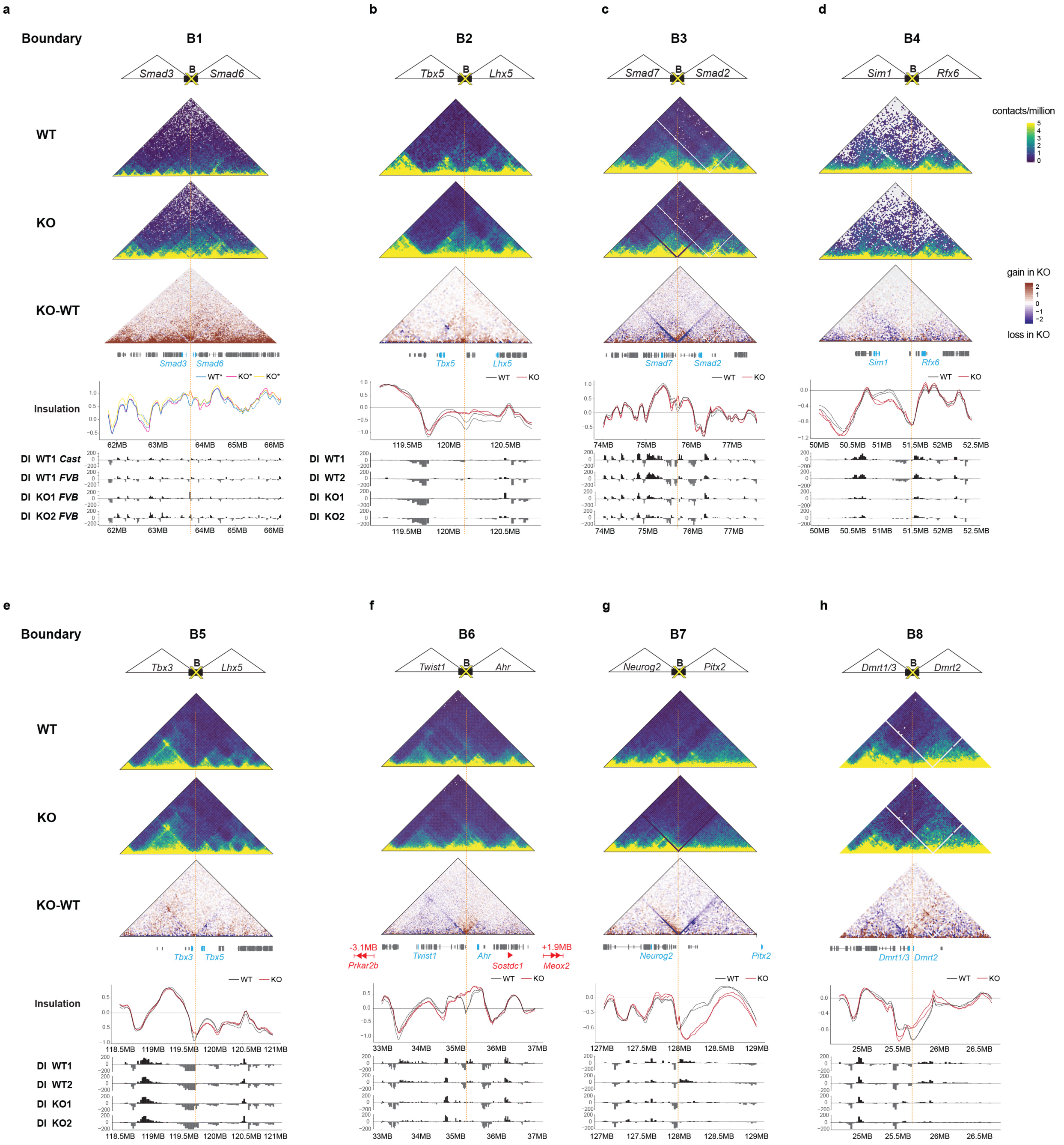


**Supplemental Figure 4. TAD boundary deletions result in abnormal TAD architecture. a-h)** Hi-C interaction maps of TAD boundary loci B1-8 showing interaction frequencies in representative wild- type (WT) and knockout (KO) mouse liver tissue for all TAD boundary loci deleted in this study. For locus B1, the WT represents the wild-type allele in Cast background and KO represents the deletion allele in FVB background from an animal heterozygous for the TAD boundary deletion. Insulation profiles and Directionality Index (DI) for two wild-type and two homozygous mutants, i.e., two biological replicates for each of the respective TAD boundary KO lines are shown for all TAD boundary loci. For locus B1, the WT* corresponds to the wild-type alleles in a mixed (Cast/FVB) strain background while the KO1 and KO2 correspond to the deletion alleles in FVB background from two separate, mixed-strain heterozygous animals. f) Genes significantly downregulated (see **Supplemental Figure 5**) are shown in red. Arrowheads indicate the relative location and approximate distance in MB from the actual TAD boundary deletion B6. Genome coordinates shown are in mm10. See **Methods** for additional details.

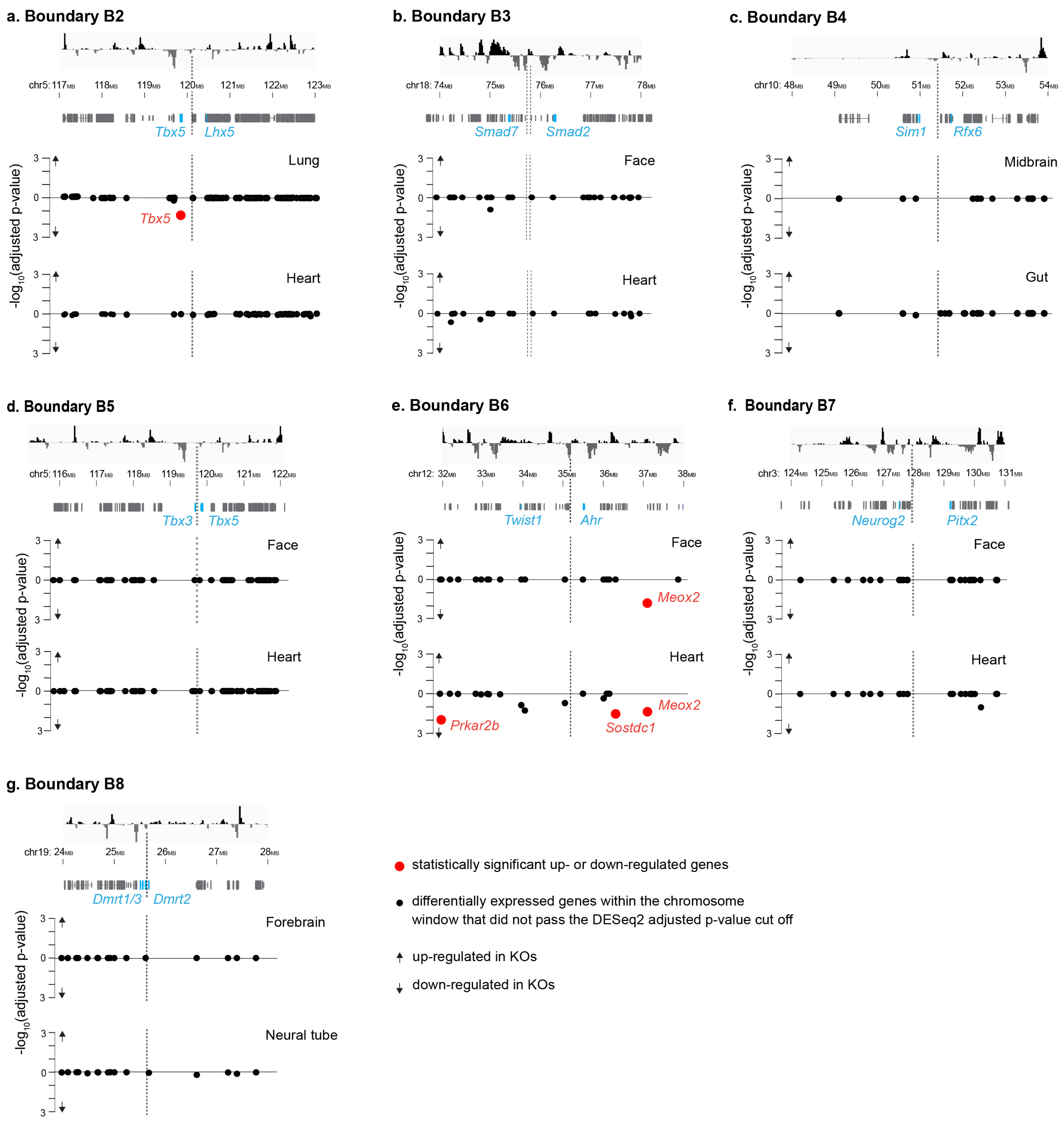


**Supplemental Figure 5. Gene expression changes in TAD boundary KOs. a-g**) Local gene expression changes in two selected tissues at E11.5 for each of the TAD boundary deletion lines. Directionality Index track along with chromosomal coordinates are shown for each locus, for reference. Gray bars show RefSeq genes in the respective regions. Genes highlighted in blue are developmentally expressed genes flanking the boundary. For gene expression plots in the lower panels, the x-axis denotes genomic location along the relevant chromosome, while the y-axis represents $\log_{10}$(adjusted p-value) and shows the specific tissues in which RNA-seq was performed. The vertical, dashed gray line indicates the position of the TAD boundary. Homozygous mutants (n=2) compared to wild-type (n=2) mice were used for each of the TAD boundary KO lines B3-8; an additional 2 samples (n=4) for each of the genotype groups were used for B2. Red points (**a, e**) indicate statistically significant up- or down-regulated genes (adjusted p-value < 0.05 using an FDR < 5%, see **Methods** for details). Genome coordinates shown are in mm10.

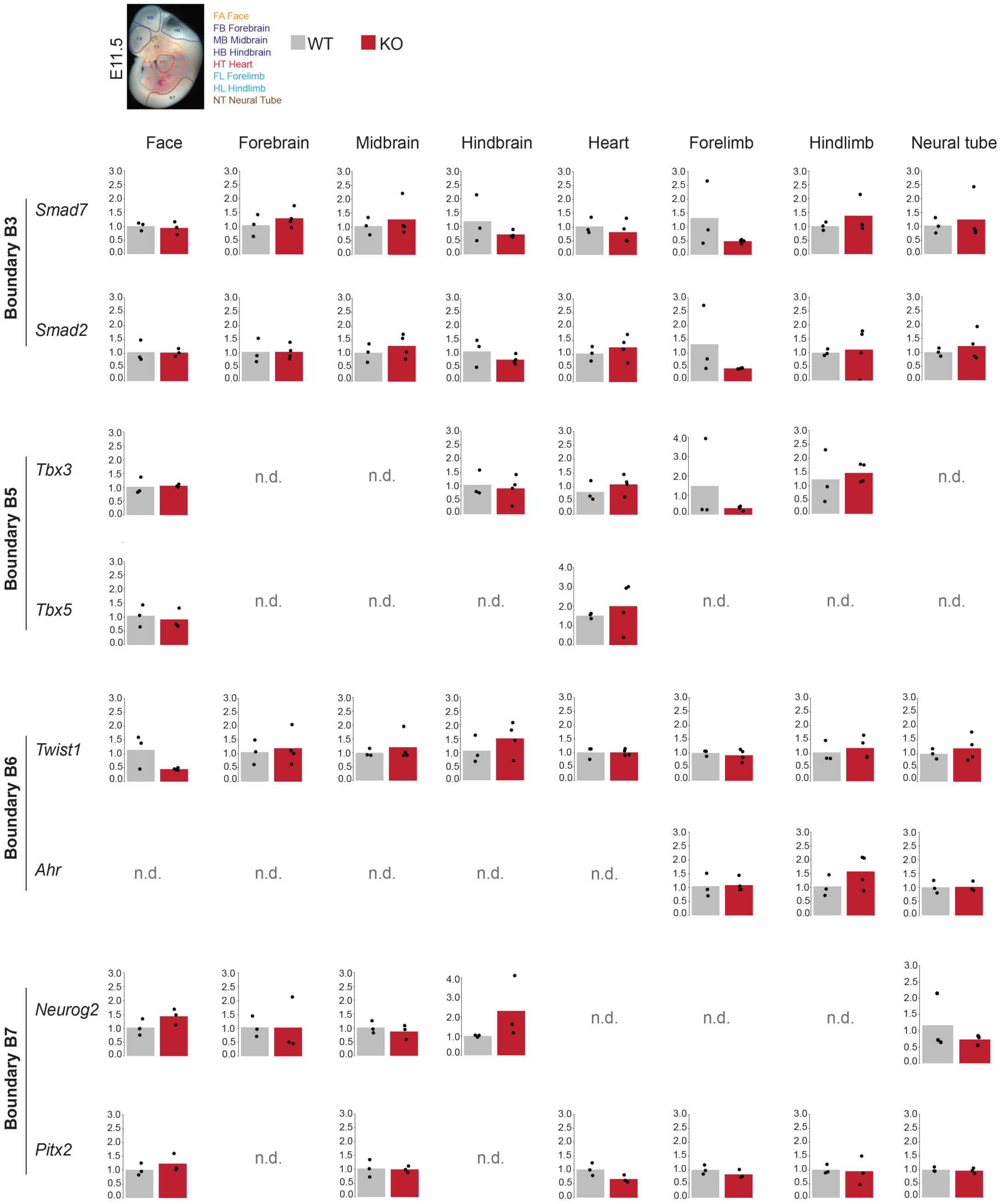


**Supplemental Figure 6. qPCR gene expression for select genes within flanking TADs**. Image shows a wild-type E11.5 mouse embryo for illustrative purpose outlining the tissue dissection outlines. Quantitative real-time PCR results for TAD boundary KO lines B3 and B5-7 showing normalized gene expression (y-axis). qPCR results from a panel of tissues (face, forebrain, midbrain, hindbrain, heart, forelimb, hindlimb and neural tube) from homozygous mutants (KO, red bars) and wild-type (WT, grey bars) embryos at E11.5 reveals lack of significantly up- or downregulated transcript levels in selected developmentally important genes, each present in a TAD adjacent to the deleted boundary. Bars denote average gene expression levels, while black dots show individual samples (n= at least 3) for each of the KO and WT groups. p-value=0.05*,* from an unpaired, two-tailed *t*-test was considered as statistically significant. n.d.=not determined. See **Supplementary Data 6** and **Methods** for additional details.

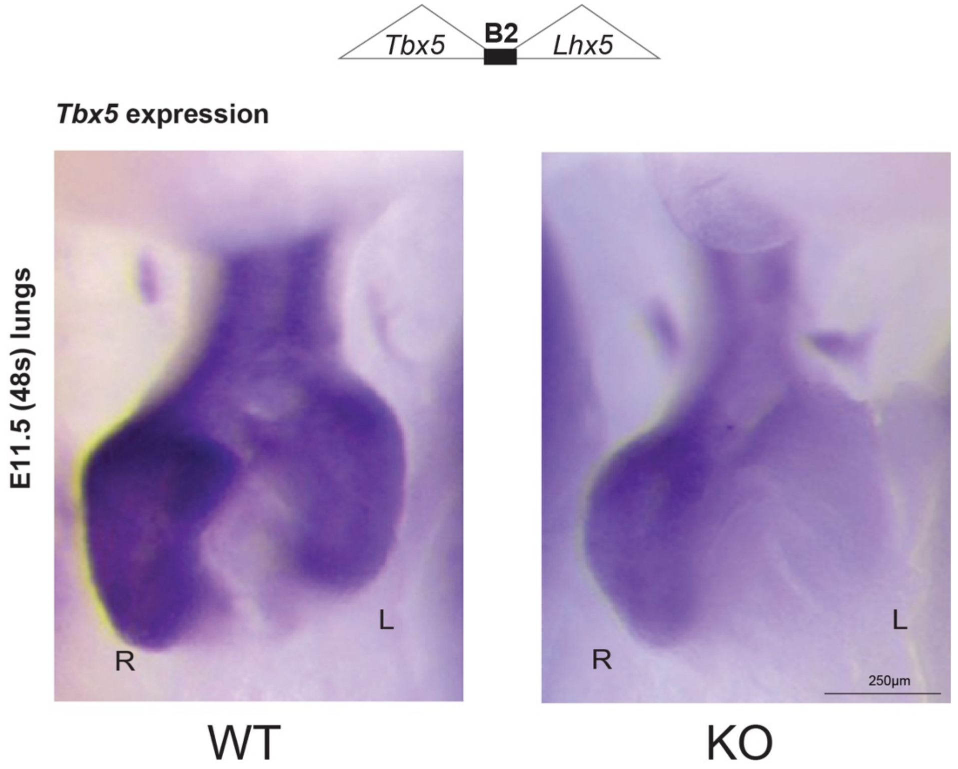


**Supplemental Figure 7. *Tbx5* expression is downregulated in TAD boundary B2 homozygous-null mutants.** *In situ* hybridization results for *Tbx5* assessed at E11.5 or 48 somite-stage, for representative wild-type and comparable TAD boundary B2 homozygous-null mutants showing reduced expression of *Tbx5* in the mutants. The gene expression differences are highly distinct in the left lung. Scale bar, 250µm.

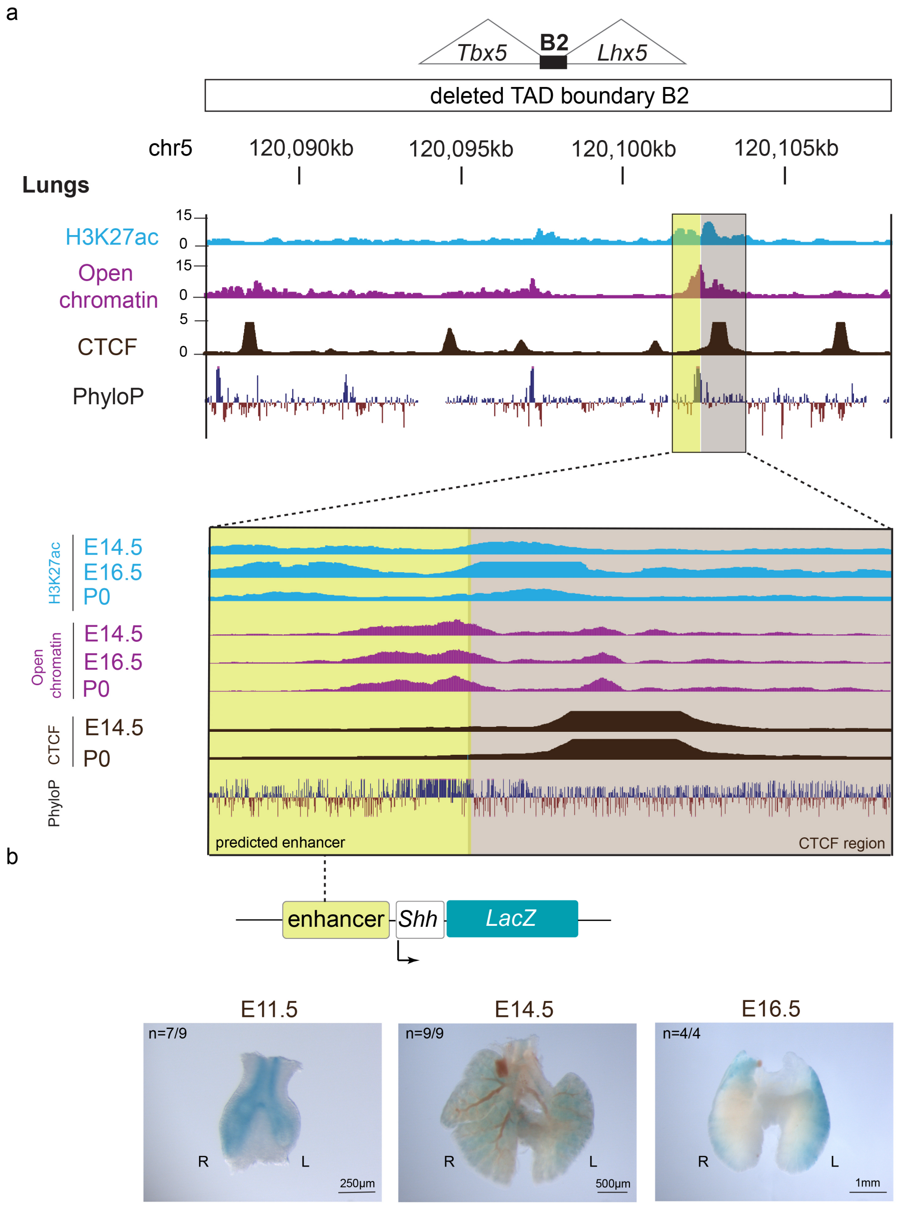


**Supplemental Figure 8. Regulatory element within TAD boundary B2 functions as lung-specific enhancer. a)** Upper panel shows the 21 kb span of the TAD boundary B2, in the vicinity of *Tbx5*, with histone modification, open chromatin data from ATAC-seq and placental conservation (PhyloP scores) from publicly available ENCODE3 data and the UCSC Genome Browser. Tracks for CTCF-bound regions are from ChIP-seq data summarized in Table 1. All tracks shown are for mouse lung tissue. The next panel shows a magnified view of the predicted lung enhancer (highlighted yellow, 852 bp), along with its adjacent insulating CTCF-bound region (highlighted beige, 1.3 kb). H3K27ac bound regions (blue), open chromatin regions from ATAC-seq assays (purple) and CTCF-bound regions (dark brown) are shown along with the respective mouse developmental stages indicated adjacent to the tracks. **b)** Cartoon shows that the predicted enhancer was cloned upstream of the *Shh* promoter-*LacZ* for transgenic enhancer-reporter assay, described in detail in Methods. Brightfield images of the lungs are shown at E11.5, 14.5 and 16.5 respectively, for the predicted enhancer element driving positive β-galactosidase staining in the lungs at all stages examined. n, independent biological replicates with reproducible results out of the total transgenic embryos obtained. Genome coordinates shown are in mm10. Scale bars, 250µm (E11.5), 500µm (E14.5), and 1mm (E16.5).

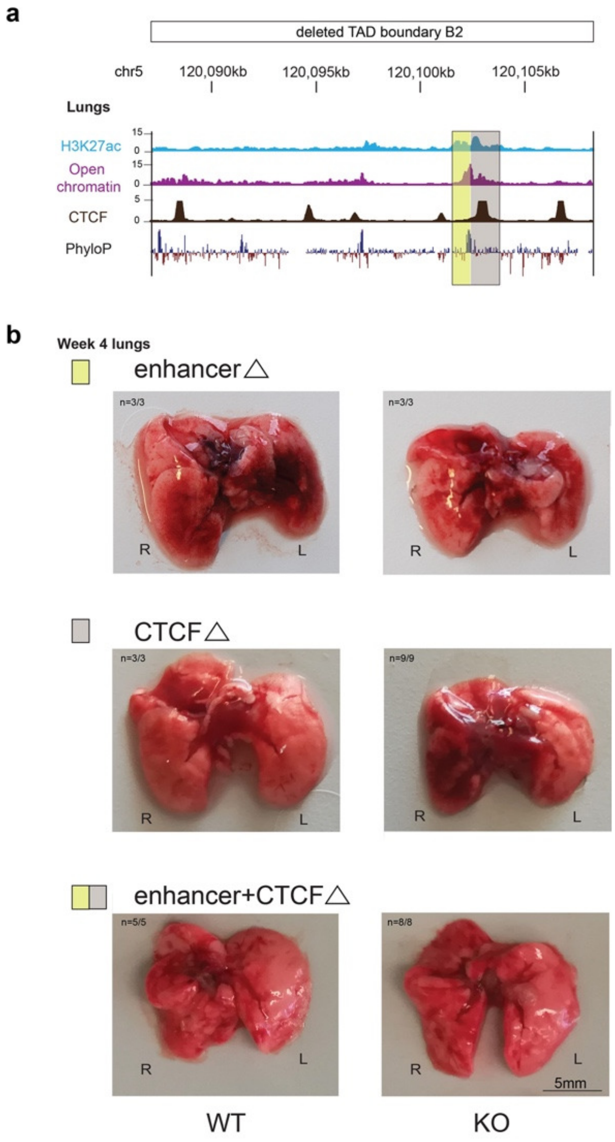


**Supplemental Figure 9. Deletions of predicted lung enhancer and insulator sequences within the TAD Boundary B2 show no obvious defects in external lung morphology. a)** Upper panel shows the 21 kb span of the TAD boundary B2, in the vicinity of Tbx5, with histone modification, open chromatin data from ATAC-seq and placental conservation (PhyloP scores) from publicly available ENCODE3 data and the UCSC Genome Browser. All tracks shown are for mouse lung tissue. See Supplemental Figure 12, Supplementary Data 8 and 9, and Methods for details. The deleted lung enhancer (861bp), along with its adjacent insulating CTCF-bound region (1.14kb) are highlighted in yellow and beige respectively. **b)** Lungs retrieved at 4-weeks from wild-type and littermate homozygous-null mice for the respective deletions of the lung enhancer, adjacent insulating CTCF-bound region, and lung enhancer+CTCF-bound region, as described in (a). No obvious differences in external lung morphology are observed in the respective mutant-control pairs for any of the intra-TAD boundary B2 deletion sequences. n, independent biological replicates with reproducible results out of the total mice assessed for a given genotype for the specific deletion. Genome coordinates shown are in mm10. L, left lung; R, right lung. Scale bar, 5mm.

**a**

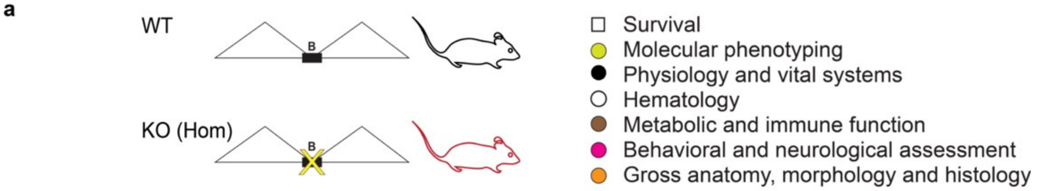


**b**

| | Age | B1 | B2 | B3 | B4 | B5 | B6 | B7 | B8 | Test |
|---|---|---|---|---|---|---|---|---|---|---|
| Longitudinal | E11.5 | X | ✓ | ✓ | ✓ | ✓ | ✓ | ✓ | ✓ | Gene Expression (RNA Seq, qRT-PCR) (Molecular phenotyping) |
| Longitudinal | Week 04 | ✓ | ✓ | ✓ | ✓ | ✓ | ✓ | ✓ | ✓ | Survival (Survival) |
| Longitudinal | Week 04 | X | ✓ | ✓ | ✓ | ✓ | ✓ | ✓ | ✓ | Body Weight, weeks 4-16 (Physiology and vital systems) |
| Longitudinal | Week 08 | ✓ | ✓ | ✓ | ✓ | ✓ | ✓ | ✓ | ✓ | Chromatin Conformation HiC (Molecular phenotyping) |
| Longitudinal | Week 08 | X | X | ✓ | X | ✓ | ✓ | ✓ | X | Open Field (Behavioral and neurological assessment) |
| Longitudinal | Week 08 | X | X | ✓ | X | ✓ | ✓ | ✓ | X | Combined SHIRPA Dysmorphology (Physiology and vital systems) |
| Longitudinal | Week 08 | X | X | ✓ | X | ✓ | ✓ | ✓ | X | Grip Strength (Behavioral and neurological assessment) |
| Longitudinal | Week 09 | X | X | ✓ | X | ✓ | ✓ | ✓ | X | Hole Poke (Behavioral and neurological assessment) |
| Longitudinal | Week 09 | X | X | ✓ | X | ✓ | ✓ | ✓ | X | Light/Dark Box (Behavioral and neurological assessment) |
| Longitudinal | Week 10 | X | X | ✓ | X | ✓ | ✓ | ✓ | X | Acoustic Startle/Pre-pulse Inhibition (Behavioral and neurological assessment) |
| Longitudinal | Week 10 | X | X | ✓ | X | ✓ | ✓ | ✓ | X | Gait Analysis (Physiology and vital systems) |
| Longitudinal | Week 11 | X | X | ✓ | X | ✓ | ✓ | ✓ | X | Fear Conditioning (Behavioral and neurological assessment) |
| Longitudinal | Week 12 | X | X | ✓ | X | ✓ | ✓ | ✓ | X | Electrocardiogram (Physiology and vital systems) |
| Longitudinal | Week 13 | X | X | ✓ | X | ✓ | ✓ | ✓ | X | Intraperitoneal Glucose Tolerance Test (Metabolic and immune function) |
| Longitudinal | Week 14 | X | X | ✓ | X | ✓ | ✓ | ✓ | X | Auditory Brain Response (2F+2M) (Physiology and vital systems) |
| Longitudinal | Week 14 | X | X | ✓ | X | ✓ | ✓ | ✓ | X | X-Ray (5F+5M) (Physiology and vital systems; Gross anatomy, morphology and histology) |
| Longitudinal | Week 14 | X | X | ✓ | X | ✓ | ✓ | ✓ | X | Body Composition (lean/fat) (Physiology and vital systems; Gross anatomy, morphology and histology) |
| Longitudinal | Week 15 | X | X | ✓ | X | ✓ | ✓ | ✓ | X | Eye Morphology (Gross anatomy, morphology and histology) |
| Longitudinal | Week 15 | X | X | ✓ | X | ✓ | ✓ | ✓ | X | Hot Plate (Behavioral and neurological assessment) |
| Terminal | Week 04 / Week 16 | X | X | ✓ | X | ✓ | ✓ | ✓ | X | Hematology (Hematology) |
| Terminal | Week 04 / Week 16 | X | X | ✓ | X | ✓ | ✓ | ✓ | X | Clinical Chemistry (Metabolic and immune function) |
| Terminal | Week 04 / Week 16 | X | ✓ (Week 04) | ✓ (Week 04) | ✓ | ✓ | ✓ | ✓ | ✓ (Week 04) | Gross Pathology and Tissue Collection (2F+2M) (Gross anatomy, morphology and histology) |
| Terminal | Week 04 / Week 16 | X | ✓ (Week 04) | ✓ (Week 04) | ✓ | ✓ | ✓ | ✓ | ✓ (Week 04) | Organ Weight (Physiology and vital systems; Gross anatomy, morphology and histology) |
| Terminal | Week 04 / Week 16 | X | X | ✓ | X | ✓ | ✓ | ✓ | X | Immunophenotyping (3F+3M) (Metabolic and immune function) |
| Terminal | Week 04 / Week 16 | X | ✓ (Week 04) | ✓ (Week 04) | ✓ | ✓ | ✓ | ✓ | ✓ (Week 04) | Histopathology (2F+2M (Gross anatomy, morphology and histology) |

**Supplemental Figure 10. Comprehensive phenotyping strategy. a)** An overview of the phenotyping strategy, including broader categories of phenotypic assessment and test types. **b)** Details of tests conducted. Ages (embryonic or postnatal) at which the tests were performed in homozygous mutants and wild-type controls are depicted on the Y-axis, for the corresponding phenotyping test performed (check marks) on the specific TAD boundary deletion mutant. Instances where phenotyping test was not performed is also indicated (grey cross-marks). The names of the tests are listed using standard terminology. More details are described in the **Supplementary Data 7**, and **Methods** section.

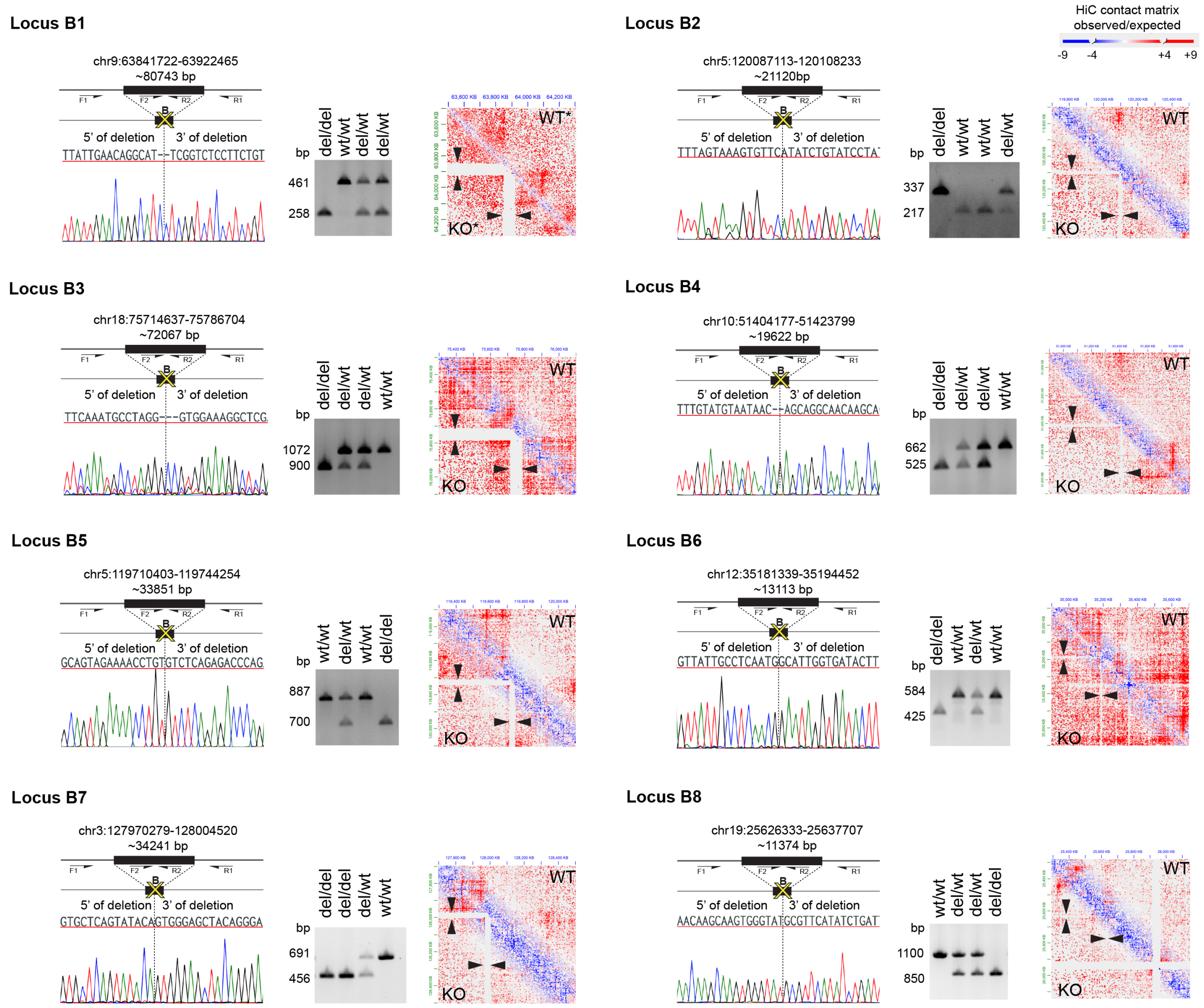


**Supplemental Figure 11. CRISPR-mediated deletion of eight TAD boundaries.** For each of the TAD boundary deletion loci B1-8, boundary regions (B) depicted as solid black bars, along with the chromosomal coordinates (mm10) and deletion span. Yellow crosses coincide with CRISPR-mediated deletion breakpoints. Sanger sequencing traces show the deletion breakpoints (indicated by the vertical dashed line) for the TAD boundary deletion alleles. Black arrows indicate forward, and reverse primers used for PCR validation. See **Supplementary Data 8** and **9** for sgRNA and PCR primer sequences. Gel images for PCR genotyping and amplicon sizes for TAD boundary deletion (del/del), heterozygous (del/wt), and wild-type (wt/wt) animals are shown. Hi-C interaction heatmaps (Juicebox output) of TADs from a wild-type and a representative TAD boundary knockout (KO) for each of the deletion lines, for the respective genomic regions is additionally shown; for deletions B2-8, KO implies readout from a homozygous TAD boundary knockout except for locus B1 where WT* and KO* implies readout from wild-type and homozygous allele respectively as obtained from a heterozygous sample. Black arrowheads indicate the region corresponding to the TAD boundary deletion in a homozygous mutant juxtaposed with the identical region from the wild-type control. Genome coordinates shown are in mm10.

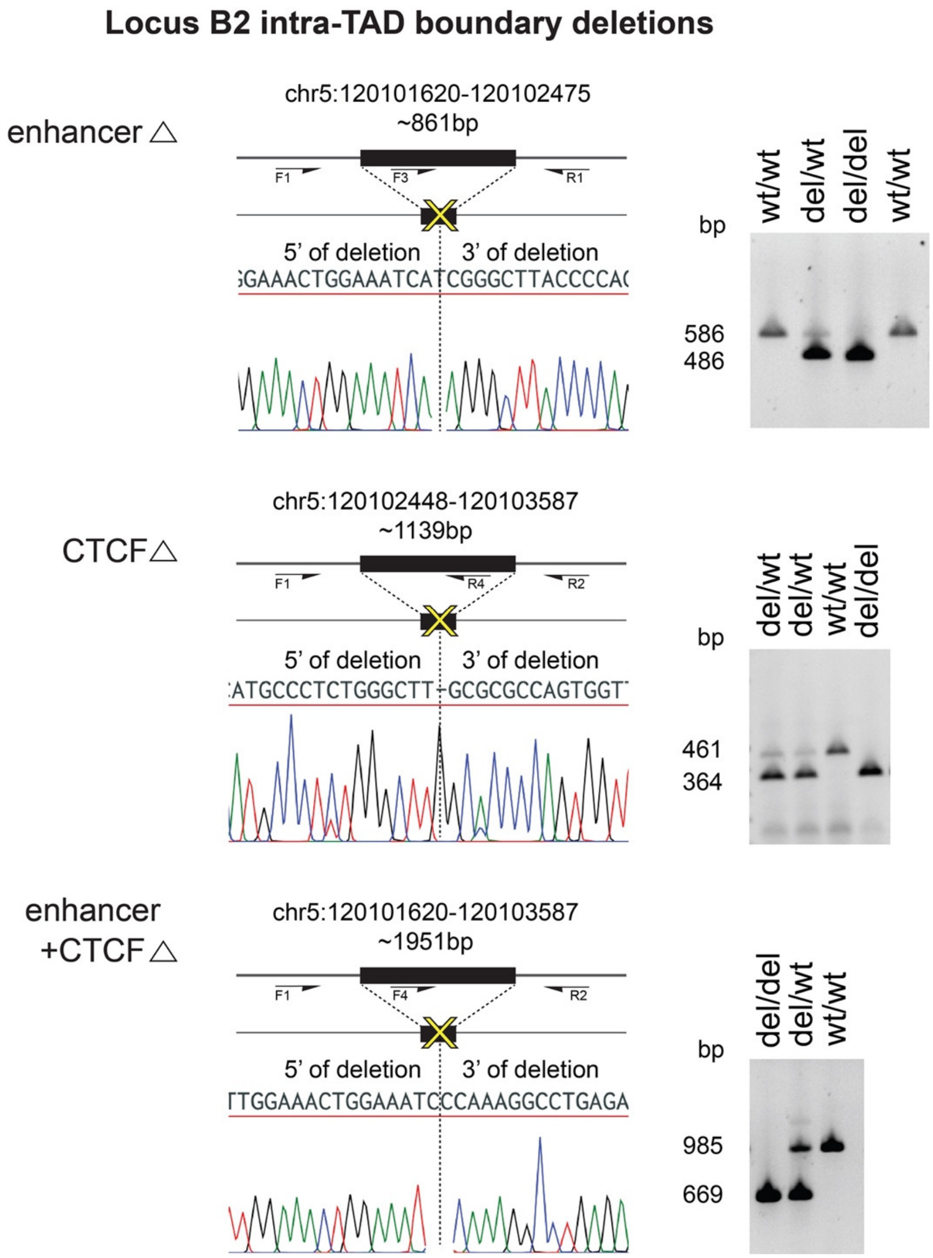


**Supplemental Figure 12. CRISPR-mediated deletion of regulatory regions within Locus B2.** For each of the deletions spanning the validated lung enhancer (enhancerΔ), its adjacent CTCF binding region (CTCFΔ) and a control region spanning both the enhancer and the CTCF site (enhancer+CTCFΔ), respective boundary deletions are depicted as solid horizontal black bars, along with the chromosomal coordinates (mm10) and deletion span. Yellow crosses coincide with CRISPR-mediated deletion breakpoints. Sanger sequencing traces show the deletion breakpoints (indicated by the vertical dashed line) for the deletion alleles. Black arrows indicate forward, and reverse primers used for PCR validation. See **Supplementary Data 8** and **9** for sgRNA and PCR primer sequences. Gel images show PCR genotyping results and amplicon sizes for the corresponding deletions for homozygous (del/del), heterozygous (del/wt), and wild-type (wt/wt) animals. Genome coordinates shown are in mm10.

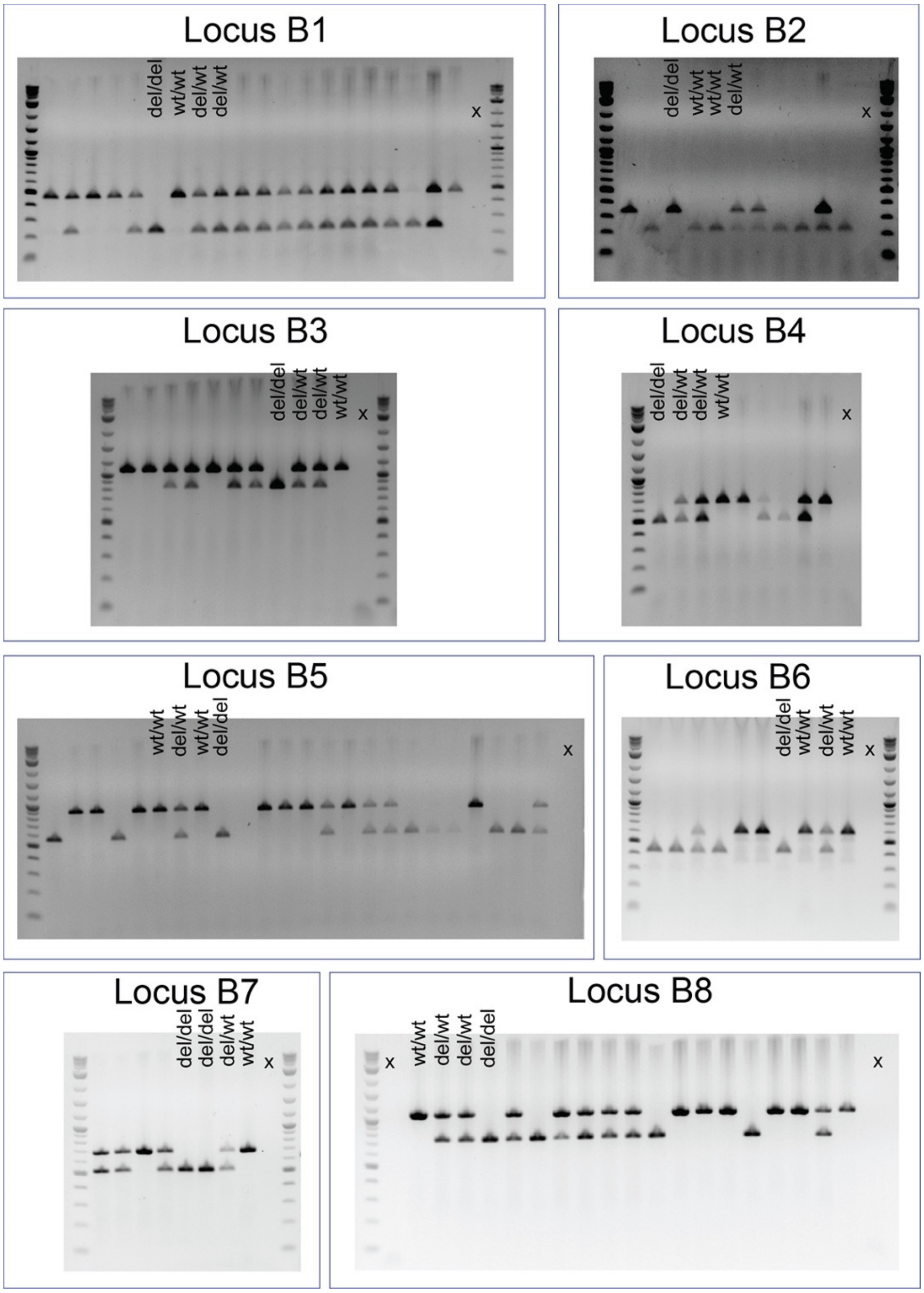


**Supplemental Figure 13. Source data for Supplemental Figure 11.** For each of the TAD boundary deletion loci 1-8, uncropped gel images are shown as source data for the gel insets shown in **Supplemental Figure 11**. A representative lane is annotated per genotype, del/del (homozygous); del/wt (heterozygous) and wt/wt (wild-type) in the context of **Supplemental Figure 11**. x, no DNA sample. Marker: 1Kb Plus DNA Ladder [ThermoFisher catalog no. 10787018].

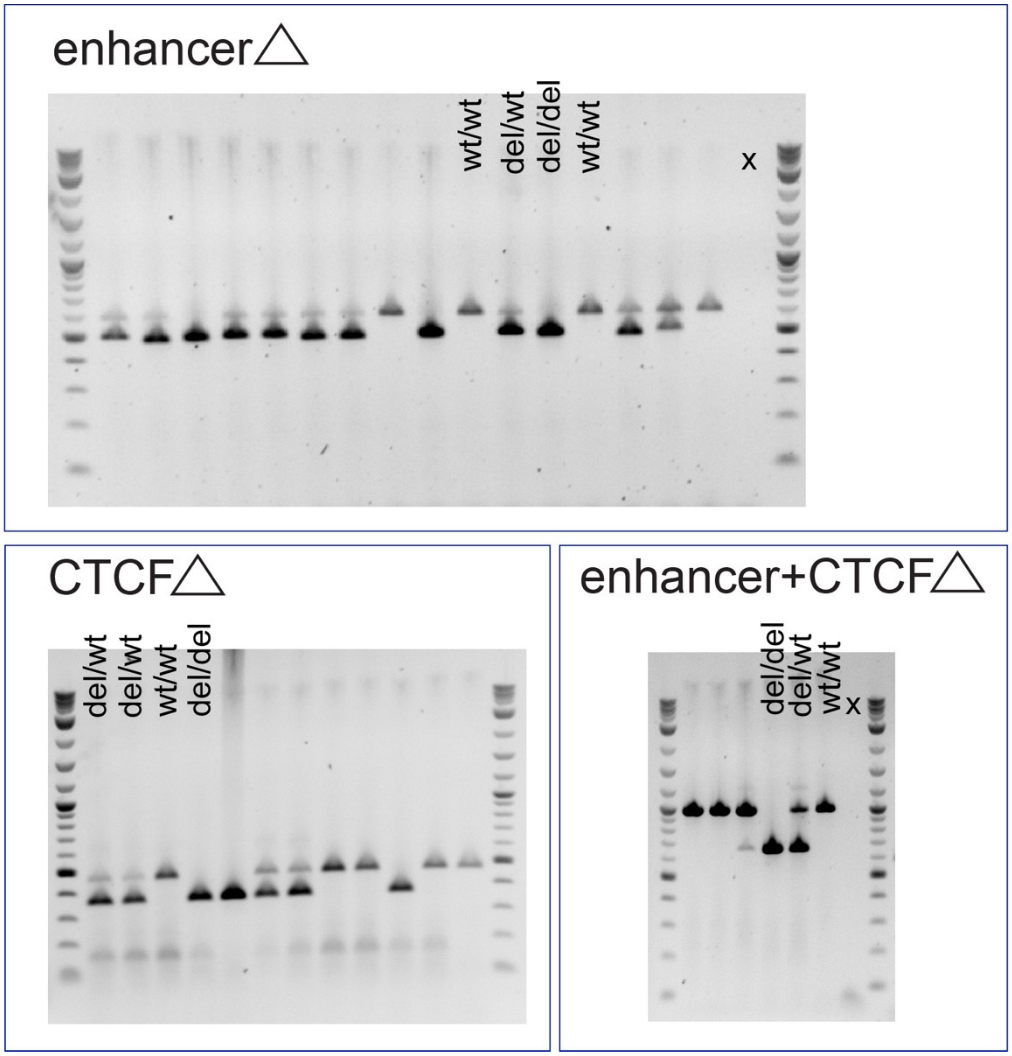


**Supplemental Figure 14. Source data for Supplemental Figure 12.** For each of the deletions spanning the validated lung enhancer (enhancerΔ), its adjacent CTCF binding region (CTCFΔ) and a control region spanning both the enhancer and the CTCF site (enhancer+CTCFΔ) within Locus B2, uncropped gel images are shown as source data for the gel insets shown in **Supplemental Figure 12**. A representative lane is annotated per genotype, del/del (homozygous); del/wt (heterozygous) and wt/wt (wild-type) in the context of **Supplemental Figure 12**. x, no DNA sample. Marker: 1Kb Plus DNA Ladder [ThermoFisher catalog no. 10787018].

## Supplementary References


1. He, P. *et al.* The changing mouse embryo transcriptome at whole tissue and single-cell resolution. *Nature* **583**, 760–767 (2020).
2. Cacheiro, P. *et al.* Human and mouse essentiality screens as a resource for disease gene discovery. *Nat. Commun.* **11**, 655 (2020).
