# Supplementary material for: Topologically associating domain boundaries are required for normal genome function
Source: Commun Biol. 2023 Apr 20;6:435. doi: 10.1038/s42003-023-04819-w (PMC10119121; doi:10.1038/s42003-023-04819-w)
Supplement: Supplementary file 2 — Description Of Additional Supplementary Files [file 42003_2023_4819_MOESM2_ESM.pdf]

Description of Additional Supplementary Files

File name: Supplementary Data 1
Description: ChIP-seq data used for correlation-based computational analysis and prioritization of TAD boundaries for deletion *in vivo*.

File name: Supplementary Data 2
Description: Correlation analyses and CTCF cluster scores used in the prioritization of TAD boundary loci for deletion *in vivo*.

File name: Supplementary Data 3
Description: Locus features of the TAD boundaries selected for individual deletions in this study.

File name: Supplementary Data 4
Description: Mendelian segregation data.

File name: Supplementary Data 5
Description: Statistically significant differentially expressed genes across select tissues in homozygous TAD boundary mutants versus wild-type embryos at E11.5.

File name: Supplementary Data 6
Description: qPCR source data.

File name: Supplementary Data 7
Description: Summary results from comprehensive phenotyping of adult mice in this study.

File name: Supplementary Data 8
Description: TAD boundaries and intra-TAD Boundary B2 sequences targeted for deletion in vivo and corresponding CRISPR sgRNA templates.

File name: Supplementary Data 9
Description: Primers used for screening and genotyping of TAD boundary and other loci generated using CRISPR-mediated deletions.

File name: Supplementary Data 10
Description: Availability of TAD boundary knockout mice.

File name: Supplementary Data 11
Description: qRT-PCR gene-specific primers.
