# Supplementary material for: Topologically associating domain boundaries are required for normal genome function
Source: Commun Biol. 2023 Apr 20;6:435. doi: 10.1038/s42003-023-04819-w (PMC10119121; doi:10.1038/s42003-023-04819-w)
Supplement: Supplementary file 14 — Reporting Summary [file 42003_2023_4819_MOESM14_ESM.pdf]

nature portfolio

Corresponding author(s): Axel Visel, Len Pennacchio

Last updated by author(s): 2023/02/07

Reporting Summary

Nature Portfolio wishes to improve the reproducibility of the work that we publish. This form provides structure for consistency and transparency in reporting. For further information on Nature Portfolio policies, see our Editorial Policies and the Editorial Policy Checklist.

Statistics

For all statistical analyses, confirm that the following items are present in the figure legend, table legend, main text, or Methods section.

n/a	Confirmed	
☐	☒	The exact sample size (*n*) for each experimental group/condition, given as a discrete number and unit of measurement
☐	☒	A statement on whether measurements were taken from distinct samples or whether the same sample was measured repeatedly
☐	☒	The statistical test(s) used AND whether they are one- or two-sided<br>*Only common tests should be described solely by name; describe more complex techniques in the Methods section.*
☐	☒	A description of all covariates tested
☐	☒	A description of any assumptions or corrections, such as tests of normality and adjustment for multiple comparisons
☐	☒	A full description of the statistical parameters including central tendency (e.g. means) or other basic estimates (e.g. regression coefficient) AND variation (e.g. standard deviation) or associated estimates of uncertainty (e.g. confidence intervals)
☐	☒	For null hypothesis testing, the test statistic (e.g. *F*, *t*, *r*) with confidence intervals, effect sizes, degrees of freedom and *P* value noted<br>*Give P values as exact values whenever suitable.*
☒	☐	For Bayesian analysis, information on the choice of priors and Markov chain Monte Carlo settings
☐	☒	For hierarchical and complex designs, identification of the appropriate level for tests and full reporting of outcomes
☐	☒	Estimates of effect sizes (e.g. Cohen's *d*, Pearson's *r*), indicating how they were calculated

Our web collection on statistics for biologists contains articles on many of the points above.

Software and code

Policy information about availability of computer code

Data collection	No software was used for data collection
Data analysis	All statistics were estimated, and plots were generated using the statistical computing environment R (www.r-project.org).

For manuscripts utilizing custom algorithms or software that are central to the research but not yet described in published literature, software must be made available to editors and reviewers. We strongly encourage code deposition in a community repository (e.g. GitHub). See the Nature Portfolio guidelines for submitting code & software for further information.

Data

Policy information about availability of data

All manuscripts must include a data availability statement. This statement should provide the following information, where applicable:

- Accession codes, unique identifiers, or web links for publicly available datasets
- A description of any restrictions on data availability
- For clinical datasets or third party data, please ensure that the statement adheres to our policy

The Hi-C and RNA-seq data discussed in this publication have been deposited in NCBI's Gene Expression Omnibus and are accessible through GEO Series accession number GSE172089 (Reviewer access token: yvutceuufhsprub). Additional data supporting the findings of this study are available from the corresponding authors upon reasonable request.

Human research participants

Policy information about studies involving human research participants and Sex and Gender in Research.

Reporting on sex and gender	Not applicable
Population characteristics	Not applicable
Recruitment	Not applicable
Ethics oversight	Not applicable

Note that full information on the approval of the study protocol must also be provided in the manuscript.

Field-specific reporting

Please select the one below that is the best fit for your research. If you are not sure, read the appropriate sections before making your selection.

☒ Life sciences ☐ Behavioural & social sciences ☐ Ecological, evolutionary & environmental sciences

For a reference copy of the document with all sections, see nature.com/documents/nr-reporting-summary-flat.pdf

Life sciences study design

All studies must disclose on these points even when the disclosure is negative.

Sample size	Detailed in Methods' section, also where specific information is related to the particular experimental approach used.
Data exclusions	No data was excluded from the study.
Replication	Replication attempts were successful and reproducible, numbers are provided in the figure panels/text as required.
Randomization	Detailed in Methods section, where applicable.
Blinding	Detailed in Methods section, where applicable.

Reporting for specific materials, systems and methods

We require information from authors about some types of materials, experimental systems and methods used in many studies. Here, indicate whether each material, system or method listed is relevant to your study. If you are not sure if a list item applies to your research, read the appropriate section before selecting a response.

Materials & experimental systems

n/a	Involved in the study
☒	☐ Antibodies
☒	☐ Eukaryotic cell lines
☒	☐ Palaeontology and archaeology
☐	☒ Animals and other organisms
☒	☐ Clinical data
☒	☐ Dual use research of concern

Methods

n/a	Involved in the study
☒	☐ ChIP-seq
☒	☐ Flow cytometry
☒	☐ MRI-based neuroimaging

Animals and other research organisms

Policy information about studies involving animals; ARRIVE guidelines recommended for reporting animal research, and Sex and Gender in Research

Laboratory animals	TAD boundary knockouts were performed in Mus musculus FVB and Cast strain mice. Mice across developmental stages from embryonic day 10.5 through P0, as well as mice between weeks 4-16 were used in this study. Animals of both sexes were used in the analysis. Sample size selection and randomization strategies are included in individual method sections.

Wild animals	NA. No wild animals were used in this study.
Reporting on sex	Animals of both sexes were used in the analysis. Sample size selection and randomization strategies are included in individual method sections.
Field-collected samples	NA. No field-collected samples are involved in this study.
Ethics oversight	All animal work was reviewed and approved by the Lawrence Berkeley National Laboratory Animal Welfare Committee. Mice were monitored and inspected by the Chair of the Animal Welfare and Research Committee and the head of the animal facility in consultation with the veterinary staff. The LBNL Animal Care Facility is accredited by the American Association for the Accreditation of Laboratory Animal Care (AAALAC).

Note that full information on the approval of the study protocol must also be provided in the manuscript.
